# Soft tissue tumor imaging in adults: European Society of Musculoskeletal Radiology–Guidelines 2024: imaging immediately after neoadjuvant therapy in soft tissue sarcoma, soft tissue tumor surveillance, and the role of interventional radiology

**DOI:** 10.1007/s00330-024-11242-0

**Published:** 2024-12-18

**Authors:** Iris-Melanie Noebauer-Huhmann, Joan C. Vilanova, Olympia Papakonstantinou, Marc-André Weber, Radhesh K. Lalam, Violeta Vasilevska Nikodinovska, Hatice T. Sanal, Frédéric E. Lecouvet, Ana Navas, José Martel-Villagrán, Jacky W. J. de Rooy, Jan Fritz, Koenraad Verstraete, Thomas Grieser, Pavol Szomolanyi, Snehansh Chaudhary, Luca Maria Sconfienza, Alberto S. Tagliafico, P. Diana Afonso, Omar M. Albtoush, Giacomo Aringhieri, Remide Arkun, Gunnar Aström, Alberto Bazzocchi, Rajesh Botchu, Martin Breitenseher, Danoob Dalili, Mark Davies, Milko C. de Jonge, Berna D. Mete, Jan L. M. A. Gielen, Geoff Hide, Amanda Isaac, Slavcho Ivanoski, Ramy M. Mansour, Catherine Mccarthy, Lorenzo Muntaner-Gimbernat, Paul O’Donnell, Şebnem Örgüç, Winston J. Rennie, Santiago Resano, Philip Robinson, Simone A. J. Ter Horst, Kirsten van Langevelde, Klaus Wörtler, Marita Koelz, Joannis Panotopoulos, Reinhard Windhager, Barbara J. Fueger, Maximilian Schmid, Filip M. Vanhoenacker

**Affiliations:** 1 Department of Biomedical Imaging and Image Guided Therapy, Division of Neuroradiology and Musculoskeletal Radiology, Medical University of Vienna, Vienna, Austria; 2 Department of Radiology, Clínica Girona, Institute of Diagnostic Imaging (IDI) Girona, University of Girona, Girona, Spain; 3 2nd Department of Radiology, Attikon Hospital, National and Kapodistrian University of Athens, Athens, Greece; 4 Institute of Diagnostic and Interventional Radiology, Pediatric Radiology and Neuroradiology, University Medical Center Rostock, Rostock, Germany; 5 Department of Radiology, Robert Jones and Agnes Hunt Orthopaedic Hospital, Oswestry, UK; 6 Medical Faculty, University “Ss. Cyril and Methodius”, Skopje, North Macedonia; 7 Department of Diagnostic and Interventional Radiology, University Surgical Clinic “St. Naum Ohridski”, Skopje, North Macedonia; 8 Radiology Department, University of Health Sciences, Gülhane Training and Research Hospital, Ankara, Türkiye; 9 Department of Radiology and Medical Imaging, Cliniques Universitaires Saint Luc, Institut de Recherche Expérimentale et Clinique (IREC), Institut du Cancer Roi Albert II (IRA2), Université Catholique de Louvain (UCLouvain), Brussels, Belgium; 10 Department of Radiology, Leiden University Medical Center, Leiden, The Netherlands; 11 Radiology Department, Hospital Universitario Fundación Alcorcón, Madrid, Spain; 12 Department of Imaging, Radiology, Radboud University Medical Center, Nijmegen, The Netherlands; 13 Department of Radiology, NYU Grossman School of Medicine, New York, USA; 14 Diagnostic and Interventional Radiology, Eberhard Karls University Tuebingen, University Hospital Tuebingen, Tübingen, Germany; 15 Department of Radiology, Ghent University Hospital, Ghent, Belgium; 16 Department for Diagnostic and Interventional Radiology, University Hospital Augsburg, Augsburg, Germany; 17 High Field MR Center, Department of Biomedical Imaging and Image‑Guided Therapy, Medical University Vienna, Vienna, Austria; 18 Department of Imaging Methods, Institute of Measurement Science, Slovak Academy of Sciences, Bratislava, Slovakia; 19 Oxford University Hospitals NHS Foundation Trust, Oxford, UK; 20 IRCCS Istituto Ortopedico Galeazzi, Milan, Italy; 21 Dipartimento Di Scienze Biomediche Per La Salute, Università Degli Studi Di Milano, Milan, Italy; 22 Department of Health Sciences (DISSAL), University of Genoa, Genoa, Italy; 23 Department of Radiology, IRCCS Ospedale Policlinico San Martino, Genoa, Italy; 24 Hospital Particular da Madeira, and Hospital da Luz Lisboa, Lisbon, Portugal; 25 Department of Radiology, University of Jordan, Ammam, Jordan; 26 Academic Radiology, Department of Translational Research and New Technologies in Medicine and Surgery, University of Pisa, Pisa, Italy; 27 Ege University Medical School (Emeritus), Izmir, Türkiye; 28 Star Imaging Center, Izmir, Türkiye; 29 Department of Immunology, Genetics and Pathology (including Oncology), Uppsala University, Uppsala, Sweden; 30 Diagnostic and Interventional Radiology, IRCCS Istituto Ortopedico Rizzoli, Bologna, Italy; 31 Department of Musculoskeletal Radiology, Royal Orthopedic Hospital, Birmingham, UK; 32 Sigmund Freud Privatuniversität, Vienna, Austria; 33 Academic Surgical Unit, South West London Elective Orthopaedic Centre (SWLEOC), London, UK; 34 Department of Radiology, St. Antonius Hospital, Utrecht, The Netherlands; 35 Department of Radiology School of Medicine, Izmir Demokrasi University, Izmir, Türkiye; 36 Department of Radiology, Jessa Ziekenhuis, Campus Virga Jesse, Hasselt, Belgium; 37 Department of Radiology, Freeman Hospital, Newcastle Upon Tyne, UK; 38 School of Biomedical Engineering and Imaging Sciences, King’s College London, London, UK; 39 St. Erasmo Hospital for Orthopaedic Surgery and Traumatology Ohrid, Ohrid, North Macedonia; 40 Oxford University Hospitals, Oxford, UK; 41 Oxford Musculoskeletal Radiology and Oxford University Hospitals, Oxford, UK; 42 Hospital Univeritario Son Espases Balearic Islands University, Palma, Spain; 43 Royal National Orthopaedic Hospital NHS Trust | RNOH · Department of Radiology, London, UK; 44 Manisa Celal Bayar University, Manisa, Türkiye; 45 Clinical MSK Radiology, Loughborough University, Leicester Royal Infirmary, Leicester, UK; 46 Hospital Universitario Ramón y Cajal, Madrid, Spain; 47 Musculoskeletal Radiology Department Chapel Allerton Hospital, Leeds Teaching Hospitals NHS Trust, Leeds, UK; 48 NIHR Leeds Biomedical Research Centre, Leeds, UK; 49 Princess Máxima Center for Pediatric Oncology, Utrecht, The Netherlands; 50 Department of Radiology and Nuclear Medicine, University Medical Centre Utrecht, Utrecht, The Netherlands; 51 Musculoskeletal Radiology Section, Klinikum Rechts der Isar, Technical University of Munich ‑ TUM School of Medicine, Munich, Germany; 52 Department of Pathology, Medical University of Vienna, Vienna, Austria; 53 Department of Orthopaedics and Traumatology, Medical University of Vienna, Vienna, Austria; 54 Department of Biomedical Imaging and Image Guided Therapy, Medical University of Vienna, Vienna, Austria; 55 Department of Radiation Oncology, Medical University of Vienna, Vienna, Austria; 56 Department of Radiology AZ Sint Maarten Mechelen, University (Hospital) Antwerp, Antwerp, Belgium; 57 Faculty of Medicine and Health Sciences, University of Ghent, Ghent, Belgium

**Keywords:** Practice guideline, Consensus, Soft tissue neoplasms, Sarcoma (Soft tissue), Diagnostic imaging

## Abstract

**Objectives:**

An update of the first European Society of Musculoskeletal Radiology (ESSR) consensus on soft tissue tumor imaging in 2015 became necessary due to technical advancements, further insights into specific entities, and the revised WHO classification (2020) and AJCC staging system (2017). The third part of the revised guidelines covers algorithms and techniques beyond initial imaging: (1) Imaging after neoadjuvant therapy in soft tissue sarcoma, (2) sarcoma surveillance, and (3) special aspects, including surveillance of non-malignant entities and the role of interventional radiology.

**Materials and methods:**

A validated Delphi method based on peer-reviewed literature was used to derive consensus among a panel of 46 specialized musculoskeletal radiologists from 12 European countries. Statements that had undergone interdisciplinary revision were scored online by level of agreement (0 to 10) during two iterative rounds that could result in either ‘group consensus,’ ‘group agreement,’ or ‘lack of agreement.’

**Results:**

The three sections contain 47 statements with comments. Group consensus was reached in 91.5%, group agreement in 6.4%, lack of agreement in 2.1%. In sarcoma, imaging immediately after neoadjuvant therapy is pivotal for determining the therapy effects and for resection-planning; surveillance should include imaging at fixed grade- and type-dependent intervals. In general, MRI is the method of choice for loco-regional surveillance of soft tissue sarcomas, and chest CT to assess metastatic disease. Interventional radiology has a role, especially in oligometastatic disease, palliative tumor control and local recurrences.

**Conclusion:**

Strategies for standardized soft tissue tumor imaging regarding therapy control, surveillance, and useful interventional procedures are provided.

**Key Points:**

***Question*** *An ESSR consensus update on soft tissue tumor imaging regarding surveillance became necessary due to technical advancements, further entity-specific insights, and revised WHO- and AJCC-classifications.*

***Findings*** *Imaging immediately after neoadjuvant therapy in soft tissue sarcoma is pivotal. Post-therapeutic surveillance should include imaging at regular intervals, stratified for tumor grade and type.*

***Clinical relevance*** *The updated ESSR soft tissue tumor imaging guidelines aim to provide best practice expert consensus for standardized imaging, to support radiologists in their decision-making, and to improve examination comparability, both in individual patients and in future studies on individualized strategies.*

## Introduction

The updated guidelines for imaging of soft tissue tumors of the European Society of Musculoskeletal Radiology (ESSR) aim to provide best practice expert consensus recommendations for standardized imaging algorithms, techniques and reporting in soft tissue tumors of adults.

Since the first consensus in 2015 [1], technical advancements, further insights into specific entities, the revised WHO classification (2020) [2] and a new version of the American Joint Committee on Cancer (AJCC) staging system (2017) [3] made an update necessary [4]. A Delphi process [5], evidence based on current literature where possible, enables consensus on complex problems among a panel of experts [6] and has been used by several ESSR guidelines recently [7], including primary local imaging of soft tissue tumors [8], whole body staging in sarcoma, and non-malignant entities requiring special algorithms [9].

This part of the recommendations is intended to support radiologists who assess the effects of treatment and perform imaging for surveillance of soft tissue tumors. Choosing adequate treatment for soft tissue tumors is complicated by the fact that sarcomas are rare and consist of numerous histological types and subtypes. This leads to significantly different behavior and prognosis, which is also influenced by the location, depth, size and grade of the tumor [10]. Response assessment is critical in tumors undergoing neoadjuvant therapy as it provides a basis for planning subsequent treatment. Once the initial treatment is terminated, an individualized surveillance strategy must be chosen. There has been considerable debate about the role of imaging in soft tissue tumor follow-up, which we also address in our article. A short section of this paper highlights the role of interventional radiology. Standardization of imaging is especially important for comparability of serial examinations in the individual soft tissue tumor patient, and this may also facilitate multicenter studies aiming to individualize patient care.

## Materials and methods

A validated Delphi method on the base of peer-reviewed literature, as has been described elsewhere [8], was used to derive consensus among a panel of 46 specialized tumor subcommittee radiologists of the ESSR, from 12 European countries. Institutional review board approval was not required for this consensus as patients were not involved. Statements were developed with comments based on the current literature by search on PubMed and the Cochrane Library and validated by two orthopedic tumor surgeons, a specialized sarcoma pathologist, a radiation oncologist, and a radiologist with special expertise in radiology of chest and abdomen and second specialization in nuclear medicine. For each statement, the panel members scored their level of agreement by using an online questionnaire (Google Forms®) [11]. Suggestions for adjustments were incorporated for the consecutive questionnaire round either as an alternative or an optimization of the statement. In three personal meetings, open questions and comments were discussed. The scores ranged from 0 to 10, with 10 being the highest grade of agreement. Minimum statement scoring required a median of at least 8 and an interquartile range of less than 4. For the statements which fulfilled these criteria, the level of agreement was calculated. “Group consensus” was defined as at least 80% of voters scoring at least 8, “Group agreement” was defined as 67–79% of voters scoring at least 8. “Lack of agreement” was assigned if the previous conditions were not met. After round 2 the rating was terminated.

## Results

This article contains three sections, with 47 statements overall. After round 2, group consensus was reached in 43/47 statements (91.5%), group agreement was achieved in 3/47 statements (6.4%), and lack of agreement in 1/47 statements (2.1%).

The sections included (1) Imaging immediately after neoadjuvant therapy in soft tissue sarcoma, covering imaging algorithms, parameters and reports (10 statements, eight of them with group consensus, one with group agreement and one with lack of agreement), (2) soft tissue sarcoma surveillance (26 statements, 26/0/0, respectively), (3) special aspects, including surveillance of non-malignant entities, and the role of interventional radiology (11 statements, 9/2/0, respectively). Statements and their level of agreement are provided in Tables 1–3.

**Table 1:** Imaging immediately after neoadjuvant therapy in soft tissue sarcoma: statements

1.1 Clinical situation, aim of imaging	Median, IQR (difference (interval)), Level of agreement
- The aim of imaging following the start of neoadjuvant therapy is identifying viable tumor within the entire tumor mass and identifying changes compared with the baseline imaging studies performed before biopsy and start of neoadjuvant therapy. This is pivotal in planning resection and in determining the effect of neoadjuvant therapy. In addition, this may have an impact on decisions concerning (neo)adjuvant therapy.	10; 0 (10–10); 97%

1.2 Imaging modalities and algorithm	
**1.2.1 Timepoint to assess the effect of neoadjuvant therapy**	
- Unless there is clinical suspicion of tumor progression, imaging to assess the effect of neoadjuvant therapy should be done after termination of neoadjuvant therapy and as close as possible to the moment of resection. Especially when radiotherapy has been used, imaging should be done 4–6 weeks after termination of radiotherapy.	10; 1 (9–10); 97%
**1.2.2. Type of imaging**	
- As the goal of the various types of neoadjuvant therapy is reduction of viable tumor tissue, the type of imaging required is not dependent on the type of neoadjuvant therapy given. Multiparametric imaging combining MRI^a^ (angiogenesis, perfusion, permeability, cell density) and [^18^F]FDG-PET/CT (glucose metabolism) can be used to detect viable tumor and therapy-induced changes based on a combination of morphologic and functional imaging.	10; 1 (9–10); 93%
- There is no role for radiography, Tc99m bone scan, or image-guided biopsy in monitoring the effect of neoadjuvant therapy.	10; 1 (9–10); 83%
- The analysis of functional imaging parameters is moving from the use of descriptive semantic features (vascularity, cell density, glucose metabolism, hypoxia, pH) to radiomics which uses high dimensional semantic and agnostic (quantification of voxel, intervoxel, or pattern values) data.	9; 3 (7–10); 66%

1.3 Imaging parameters and report	
**1.3.1 MRI**	
- The MR protocol consists of morphologic and functional components. The morphologic part of the acquisition protocol is the same as the initial diagnostic MRI protocol^c^. The functional part, consisting of dynamic contrast-enhanced MRI and diffusion-weighted MRI, needs to be done not only in the follow-up protocol but also in the initial diagnostic protocol, as changes in functional parameters facilitate response assessment.	10; 2 (8–10); 86%
- For MRI scans performed during and after neoadjuvant treatment, the same findings need to be described in the report as at baseline^b,c^.	10; 2 (8–10); 86%
- For MRI scans performed during and after neoadjuvant treatment, additionally, after neoadjuvant therapy specific findings need to be mentioned regarding treatment response and re-evaluation of resectability. Specifically this regards location and size of viable residual tumor, changes in tumor volume and signal intensities, enhancement and diffusion characteristics.	10; 0 (10–10); 93%
- Machine learning approaches may become applicable for segmentation and evaluation of treatment response in STS.	9; 3 (7–10); 69%
**1.3.2 PET/CT**	
- FDG-PET/CT should be performed according to the latest EANM protocol version.	10; 2 (8–10); 93%

^a^ MRI in general provides the best soft tissue contrast and serves to exactly assess the anatomic structures

^b^ See Table 1 and standardized checklist on MR report in^c^

^c^ Noebauer-Huhmann et al [8]

**Table 2:** Post-therapeutic surveillance imaging in soft tissue sarcoma: statements

2.1 Overall evidence	Median, IQR (difference (interval)), Level of agreement
- Still, there are no evidence-based recommendations for routine follow-up in surgically treated sarcomas.	9; 2 (8–10); 87%

2.2 Timeline	
**2.2.1. Follow-up intervals**	
- We would generally advocate:	10; 1 (9–10); 93%
- Baseline follow-up no earlier than 3 months post-treatment^a^.	
- In high-grade sarcoma: Year 1–3 every 3–4 months, year 4–5 every 6 months, year 6–10 annually^a^.	
- In low-grade sarcoma: Year 1–3 every 6 months, year 4–10 annually^b^.	
- In grade 1 sarcoma with initial R0 resection^c^, patient-initiated follow-up, instead of regular intervals, may be considered after year 5 in compliant patients.	10; 2 (8–10); 97%
**2.2.2. Endpoint**	
- Regular follow-up should be carried out during the first 10 years after the initial diagnosis.	10; 2 (8–10); 90%
- Regular annual follow-up should be continued longer than 10 years in patients with well-differentiated (retroperitoneal) liposarcomas and myxoid liposarcoma.	10; 1 (9–10); 90%
- In case of recurrence, the surveillance algorithm should restart.	10; 1 (9–10); 93%

2.3 Modalities	
**2.3.1. Role of imaging**	
- The inclusion of Imaging in follow-up is necessary, especially in high-grade STS.	10; 1 (9–10); 97%
- The ability to offer successful salvage treatment of recurrent disease supports systematic imaging surveillance and early detection of recurrence^d^.	10; 1 (9–10); 97%
- A fixed follow-up schedule for patients with STS permits timely detection of LR and metastatic disease.	10; 1 (9–10); 97%
**2.3.2. Imaging modalities in general**	
- MRI is the method of choice for local and loco-regional surveillance of soft tissue sarcomas.	10; 0 (10–10); 97%
- In sarcomas of the mediastinum, retroperitoneum and visceral sites, CT may be indicated instead of MRI for local and loco-regional surveillance.	10; 2 (8–10); 86%
- In limb sarcomas, US represents a valuable alternative for the assessment of local recurrence if MRI is inconclusive due to artifacts, in cases where MRI is contraindicated, or in rare cases where MRI is not available.	10; 1 (9–10); 91%
- In subcutaneous low-grade lesions, and given that potential LR would be likely palpable, local surveillance with ultrasound may be considered instead of MRI.	9; 2 (8–10); 87%
- For metastatic disease, chest CT should be performed (For modified strategies in special entities and conditions please see under “Individualized strategy”).	10; 0 (10–10); 93%
- FDG PET/CT can be a useful problem-solving tool if another study is equivocal.	10; 1 (9–10); 83%
**2.3.3. Imaging parameters**	
- Local MRI:	
- The FOV should cover the whole surgical/post-therapeutic region.	10; 0 (10–10); 97%
- One anatomic landmark should be visible.	10; 0 (10–10); 97%
- Same sequence parameters as in primary diagnosis can be used, except for sites where modifications are required to reduce artifacts from metallic hardware.	10; 0 (10–10); 100%
- If possible/not contraindicated, contrast agent should be used.	10; 1 (9–10); 87%
- Whole body MRI:	
- For surveillance, the parameters of primary staging can be used.	10; 1 (9–10); 93%

2.4. Individualized surveillance strategy	
**2.4.1. Myxoid liposarcoma (MLS)**	
- For the detection of metastases, WB-MRI is recommended (for local surveillance, additional dedicated local MRI is recommended).	10; 2 (8–10); 83%
- For the detection of metastases, in year 0–2, chest CTs are recommended every 3 months, followed by chest radiographs every 6 months up to year 5 thereafter.	10; 2 (8–10); 100%
**2.4.2. Other entities which require specific follow-up imaging strategies**	
- Alveolar soft part sarcoma, Angiosarcoma, epithelioid sarcoma, clear cell sarcoma, rhabdomyosarcoma, leiomyosarcoma, undifferentiated pleomorphic sarcoma, retroperitoneal (well-/) de-differentiated liposarcoma^e^.	10; 1 (9–10); 97%

2.5. Other points that should be considered in surveillance of soft tissue tumors	
- Follow-up imaging should always be compared with previous images (especially those of the primary tumor, the baseline post-therapeutic images, as well as the most recent previous study). The study should ideally be performed on the same scanner, and the previous examination should be available (for copying sequence planes and parameters) and for comparative reading.	10; 1 (9–10); 97%
- Reports should contain the parameters that have been described for primary imaging (see there).	10; 1 (9–10); 97%
- Patients should be included by adequate information and encouragement to participate in the surveillance process^e^.	10; 1 (9–10); 100%

^a^ After resection or adjuvant therapy, whatever comes latest

^b^ For modified strategies (intervals and modalities) in special entities and conditions please see under “Individualized strategy”

^c^ R0 resection indicates a microscopically margin-negative resection, in which no gross or microscopic tumor remains in the primary tumor bed

^d^ For exceptions depending on sarcoma subtype, and modifying factors such as patient condition, please see below

^e^ Details are provided in the additional electronic material

**Table 3:** Special aspects: statements

3.1. Non-malignant entities that require imaging for therapy control	Median, IQR (difference (interval)), Level of agreement
- Desmoid fibromatosis, lipoblastoma, inclusion body fibromatosis, calcifying aponeurotic fibroma, Gardner fibroma require imaging for the control of therapy (among other entities, such as superficial fibromatosis or tenosynovial giant cell tumor).	10; 1 (9–10); 93%

3.2. Surveillance algorithms for non-malignant entities	
**3.2.1. Desmoid type fibromatosis**	
- A watchful waiting approach for asymptomatic patients is recommended.	10; 1 (9–10); 93%
- MRI is preferred for surveillance. It should include T2w and contrast-enhanced sequences.	10; 1 (9–10); 87%
- A watchful waiting approach should include a first re-evaluation within 8–12 weeks.	10; 2 (8–10); 87%
- General follow-up scheme by imaging every 3 months in the first year, then every 6 months up to the fifth year, and yearly thereafter in case of stable disease.	10; 2 (8–10); 87%
**3.2.2. Surveillance in cancer predisposition syndromes**	
- Whole-body MR imaging and whole-body FDG PET/CT are useful in patients with cancer predisposition syndromes. Whole-body MR imaging is preferable since the patients are not exposed to ionizing radiation.	10; 1 (9–10); 97%
For nerve sheath tumors and atypical lipomatous tumors, please see^a^.	

3.3. Role of interventional radiology	
The role of interventional radiology is expanding in different scenarios:	
-In the case of oligometastatic disease, patients should be considered for local therapies.	10; 2 (8–10); 86%
-In the case of local recurrences, patients should be considered for local therapies.	10; 1 (9–10); 90%
-Percutaneous cryoablation can be considered in case of desmoid tumors and dermatofibrosarcoma protuberans.	10; 2 (8–10); 83%
-MR-guided high-intensity focused ultrasound can be considered for desmoid tumors.	9; 2 (8–10); 76%
-Interventional radiological procedures have a role in tumor control in a palliative setting.	9; 2 (8–10); 77%

^a^ Noebauer-Huhmann et al [9]

## Discussion

This third part of the updated ESSR consensus guidelines for soft tissue tumor imaging aims to provide feasible best practice expert state-of-the-art procedures after the initial imaging. The Delphi process was chosen as it allowed anonymous scoring [12]; few additional face-to-face-meetings proved useful for discussion of open questions regarding the procedure and of statements that had not reached consensus.

The expert panel was identical to one of the two previous guideline publications and included active representatives and soft tissue tumor imaging specialists recruited from the dedicated Musculoskeletal (MSK) tumor subcommittee of the ESSR [13]. By including specialists from twelve European countries with different national infrastructure and approaches, these consensus recommendations may help to provide feasible imaging algorithms for imaging after the initial diagnosis.

In the following paragraphs, we present a selection of the most clinically relevant statements with a short discussion (Tables 1–3; additional comments are provided online as Supplementary material).

**Section 1: Imaging immediately after neoadjuvant therapy in soft tissue sarcoma** (Table 1; for further comments please also see additional electronic material (Supplementary material)):

The aim of imaging following neoadjuvant therapy is identifying viable tumor within the entire tumor mass and identifying changes compared with the baseline imaging studies performed before biopsy and start of neoadjuvant therapy [14]. This is pivotal in planning resection and in determining the effect of neoadjuvant therapy. In addition, this may have an impact on decisions concerning (neo) adjuvant therapy [15].

### Timepoint to assess the effect of neoadjuvant therapy

Unless there is clinical suspicion of tumor progression, imaging to assess the effect of neoadjuvant therapy should be done after termination of neoadjuvant therapy and as close as possible to the moment of resection.

Up to 6 weeks following termination of radiotherapy (RTx) marked edematous and inflammatory changes adversely affect interpretation of imaging. Whenever possible imaging should therefore be scheduled after this period [16].

### Type of imaging

As the goal of the various types of neoadjuvant therapy is improvement of oncological outcome by reduction of viable tumor tissue, the type of imaging required is not dependent on the type of neoadjuvant therapy given. Multiparametric imaging combining MRI (angiogenesis, perfusion, permeability, cell density) and [^18^F]FDG-PET/CT (glucose metabolism) can be used to detect viable tumor and therapy-induced changes based on a combination of morphologic and functional imaging.

**Section 2: Post-therapeutic surveillance imaging in soft tissue sarcoma** (Table 2; for further comments please also see additional electronic material (Supplementary material)):

**Follow-up intervals general recommendations:**

- Baseline follow-up no earlier than 3 m post-treatment
- In high-grade sarcoma: Year 1–3 every 3–4 months, year 4–5 every 6 months, year 6–10 annually
- In low-grade sarcoma: Year 1–3 every 6 months, year 4–10 annually
- In grade 1 sarcoma with initial R0 resection, patient initiated follow-up, instead of regular intervals, may be considered after year 5 in compliant patients.

### Local recurrence (LR)

In high-grade sarcomas, the local recurrence rate is higher than in low-grade sarcomas [17–21]. Most early recurrences are observed in high-grade sarcomas within the first 2 to 3 years of surveillance [17]. After combined surgery and radiotherapy, more than 90% of first local recurrences are observed within the first 5 years, and all recurrences within 15 years [22]. Large (> 10 cm) sarcomas are associated with late (> 5 years) local recurrences, with recommendation of long-term follow-up [23].

### Influence of low tumor grade

Recurrence seems unlikely in low-grade sarcomas after R0 resection [24]. Low-grade tumors re-occur at a constant rate throughout follow-up [17]. While late recurrence is less frequent it may occur in low-grade STS and may manifest with higher grade [25, 26]. However, late (> 5 years) 1st local recurrence is very rare in grade I tumors [23]. Thus, long-term follow-up by imaging can be regarded as unnecessary in those patients [23].

### Metastatic/distant recurrence

Factors that are associated with metastatic recurrence are high tumor grade and tumor size > 5 cm [22]. In high-grade sarcomas, the rate of distant metastases is high in the first two years and decreases afterward [17]. Sarcomas which are classified as high-grade by the Fédération Nationale des Centres de Lutte Contre le Cancer (FNCLCC) are associated with late (> 5 years) metastatic recurrences [23]. Entities for which a higher rate of metastatic recurrence has been described are leiomyosarcoma, rhabdomyosarcoma, synovial sarcoma, epithelioid sarcoma [22], undifferentiated sarcoma and de-differentiated liposarcoma [27]. In retroperitoneal sarcomas, the entities high-grade leiomyosarcoma, solitary fibrous tumor and high-grade liposarcoma are associated with an increased cumulative incidence of distant recurrence [28]. Low-grade sarcomas rarely metastasize [17]. Repeat resections of recurrent pulmonary metastasis show a significantly better prognosis than those with only one resection [29].

### Role of imaging

The inclusion of Imaging in follow-up is necessary, especially in high-grade STS. A fixed follow-up schedule for patients with STS permits timely detection of LR and metastatic disease. In a study including soft tissue sarcomas in the extremities and trunk wall, local imaging (mainly MRI) identified a statistically significant larger number of local recurrences than clinical examination did [30]. This is in accordance with a study where surveillance by MRI detected a significant number of clinically undetectable LRs (11% (34/325)), especially for LRs in the thigh or buttock, small LRs or LRs without mass formation [31]. In another study, about one-third of LRs were detected by routine imaging only [32]. However, the study did not identify the factors (such as patient, tumor, or therapeutic characteristics) that could define a subgroup of patients that are more or less likely to benefit from surveillance by imaging [32]. A recent study by Koenig et al also found that 30% of LRs were clinically inapparent. These clinically inapparent LR were smaller (mean volume 7.0 ± 1.5 cm^3^ vs. 71.9 ± 15.7 cm^3^) than clinically detectable LR [33]. Another study observed that 46 of 87 patients with LR of a soft tissue sarcoma could have been diagnosed earlier with routine cross-sectional imaging (only one of those 46 with pelvic sarcoma) [34]. In deep sarcomas, LR are often clinically undetectable [27]. In extremity STS post-surgery and RTx, 1 of 11 local recurrences in a study of 114 patients was clinically undetectable and only revealed by MRI [35]. In another study of 124 patients, 2 of 11 local recurrences of limb sarcoma were only seen by MRI; both after R1 resection, while the authors also observed false positive cases [36]. The median delay between initial surgery and detection of LR was shorter when LR was identified by imaging (median: 20.1 months range 5.3–35.7) than by clinical examination (median: 28.6 months; range 2.0–52.4) [27]. In clinically undetectable LRs, patients with MRI-detected LR showed a (non-significant) trend toward better survival [31].

### Imaging modalities in general for local and loco-regional surveillance

MRI is the method of choice for local and loco-regional surveillance [31, 32, 37–39]. In sarcomas of the mediastinum, retroperitoneum and visceral sites, CT or PET/CT may be indicated instead of MRI for local and loco-regional surveillance [40–42]. In limb sarcomas, US (by an experienced sonographer) seems to be a cost-effective primary imaging alternative for exclusion of local recurrence [43], particularly in the presence of metallic hardware [44]. However, especially in the early postoperative period, close comparison with a baseline MRI is needed [45]. In case of large metallic hardware, dual-energy CT or CT using modern iterative reconstruction algorithms of raw datasets, or PET/CT can be considered as alternative or additive to MRI [31, 46]. In MRI, the use of lower field strength and specific artifact suppression techniques can help to reduce metal artifacts [47, 48].

### Imaging modalities in general for whole-body surveillance

For pulmonary metastasis, recommended chest imaging modalities vary from chest X-ray [49] to either chest X-ray or chest CT [50, 51], to chest CT alone [52, 53]. Chest CT proved to be superior in the detection of pulmonary metastases, compared to chest X-ray [30]. While some authors did not find survival benefit from the use of chest CT [54], another study observed a longer median survival after relapse if the diagnosis of metastatic relapse was made on planned chest CT scan rather than chest X-ray [55]. FDG-PET/CT whole body can be a useful problem-solving tool if another study is equivocal, particularly in cases of suboptimal MRI because of extensive metal artifacts or where MRI is contraindicated [56–58]. Most soft tissue sarcomas, especially the more aggressive ones, are metabolically active in FDG PET/CT [59]. In the latest NCCN Guidelines, the use of CT or PET/CT in sarcomas with propensity for lymph node metastases is recommended [51, 60]. In the future, larger data set evaluations with subsequent individualized risk assessment for sarcoma patients are expected to lead to adapted surveillance strategies, including refined indication for PET/CT. An increasing availability of PET/CT scanners, the development of novel tracers, as well as entity-based tracer avidity cutoff values may lead to broader implementation of the method.

Regarding imaging parameters, the FOV should cover the whole surgical/post-therapeutic region. One anatomic landmark should be visible. In general, the US and MRI techniques that have been used for primary imaging can be used, which also facilitates comparison of the examinations. Color Doppler may help differentiate recurrent tumor mass from fibrous tissue or other non-vascularized tissue (hematoma, seroma) in the postoperative site [45], however, the lack of Doppler signal does not exclude recurrence [43].

In case of metallic hardware, lower MRI field strengths are preferred, and dedicated sequences that are optimized for minimizing susceptibility artifacts should be used [47, 48]. Although diffusion-weighted imaging is currently hampered by limited image quality, it facilitates the detection of recurrent lesions and, when evaluated in conjunction with other sequences, may increase confidence in diagnosing recurrence [61]. If possible, contrast-enhanced (CE) MRI should be used [62]. CE MRI also increases confidence in less experienced readers [63]. Dynamic contrast-enhanced MRI is useful in the differentiation of recurrent soft tissue sarcoma and post-therapeutic fibrosis [64].

#### Special entities

Because of the unconventional metastatic behavior of myxoid liposarcoma (MLS), with recurrence sites that differ from other soft tissue sarcomas (with a high proportion of extrapulmonary metastases and low incidence of pulmonary metastases), and because of its low PET-avidity [65–67] WB-MRI has been recommended for staging and follow-up [68–70]. A possible protocol contains at least coronal and axial STIR and a coronal T1w sequence [69].

**Section 3: Special aspects** (Table 3; for further comments please also see additional electronic material (Supplementary material)):

### Interventional therapy

Besides surgery and radiotherapy [71, 72], interventional radiological procedures have a role especially in the case of oligometastatic disease, in palliative tumor control and in local recurrences [73].

#### General considerations

In general, follow-up MRI should be compared with the preoperative MRI (for morphology, site, and extent of the lesion), the baseline post-therapeutic one and the most recent one, at least [1]. This is also important as a recent study by Koenig et al demonstrated a close resemblance of the MRI morphology of LRs to the initial STS, whereas the MR morphology of post-therapeutic changes in patients with suspected LRs was different [33]. Patients should be informed and their participation should be sought. This is in accordance with a study, where the vast majority felt that it was important to be included in decision-making about their follow-up regime [74].

### Limitations

As has been described earlier [8], this consensus has several limitations. The panelists came from European countries only. However, while access to modalities such as MRI and PET/CT is limited in many other parts of the world, this must be taken into account only to a certain extent. In even less privileged countries, only some parts of this consensus will be applicable at the time being. Limitations of the Delphi method have been described earlier [8], including limited possibility for open discussion. On the other hand, all critical remarks could be considered anonymously without bias by dominant participants. The process was also time-consuming, which is a major disadvantage that has been described for guidelines that contain multiple statements, such as ours [12]. As high commitment was required for several questionnaire rounds, we aimed to provide sufficient time for the experts to answer. We did not address imaging during neoadjuvant treatment. This aspect is very complex, as it is dependent both on the very variable entities and the choice of therapy, and would possibly justify a separate consensus. Finally, it should be emphasized that these guidelines reflect the current knowledge and will require further updates in future. Especially, the role of radiomics and artificial intelligence is increasing very fast.

## Conclusion

The updated ESSR guidelines on soft tissue tumor imaging for therapy control of soft tissue tumors, as well as soft tissue tumor surveillance, and special aspects, covering the role of interventional radiology, aim to provide best practice expert consensus which may support radiologists in their decision-making. Standardization may improve the comparability of serial examinations in the individual patient and may also provide databases for large data analysis aimed at developing individualized strategies.

## Supplementary information

ELECTRONIC SUPPLEMENTARY MATERIAL

## Abbreviations

AJCC: American Joint Committee on Cancer
CT: Computed tomography
ESSR: European Society of Musculoskeletal Radiology
LR: Local recurrence
MRI: Magnetic resonance imaging
MSK: Musculoskeletal
PET/CT: Positron emission tomography
RTx: Radiotherapy
US: Ultrasound
WHO: World Health Organization
