# Supplementary material for: Soft tissue tumor imaging in adults: European Society of Musculoskeletal Radiology–Guidelines 2024: imaging immediately after neoadjuvant therapy in soft tissue sarcoma, soft tissue tumor surveillance, and the role of interventional radiology
Source: Eur Radiol. 2024 Dec 18;35(6):3324–35. doi: 10.1007/s00330-024-11242-0 (PMC12081562; doi:10.1007/s00330-024-11242-0)
Supplement: Supplementary file 1 — ELECTRONIC SUPPLEMENTARY MATERIAL [file 330_2024_11242_MOESM1_ESM.pdf]

Soft tissue tumor imaging in adults: European Society of Musculoskeletal Radiology - Guidelines 2024. Imaging immediately after neoadjuvant therapy in soft tissue sarcoma, soft tissue tumor surveillance, and the role of interventional radiology

ELECTRONIC SUPPLEMENTARY MATERIAL

Section 1. Imaging immediately after neoadjuvant therapy in soft tissue sarcoma. Comments.

1.1 Clinical situation, aim of imaging:

- The aim of imaging following the start of neo-adjuvant therapy is identifying viable tumor within the entire tumor mass and identifying changes compared with the baseline imaging studies performed before biopsy and start of neo-adjuvant therapy. This is pivotal in planning resection and in determining the effect of neo-adjuvant therapy. In addition, this may have an impact on decisions concerning (neo)-adjuvant therapy.

Indication and execution of neo-adjuvant treatment is standardized according to international guidelines (for instance EMSOS) [1]. Alternatively neo-adjuvant treatment is administered in multicenter clinical trials (for instance EORTC) [2]. When there is clinical suspicion of tumor progression of during the neo-adjuvant treatment period, imaging is indicated [3]. When clinically the tumor seems to be stable or responding well to neo-adjuvant treatment, imaging is repeated after termination of neo-adjuvant therapy, prior to surgery. In the context of neo-adjuvant therapy, the aim of imaging of the primary tumor site is identifying and locating viable tumor tissue relative to the first imaging studies because this information is essential in planning surgical resection [4]. Viable tumor within the entire tumor mass must be differentiated from other components such as necrosis, hemorrhage, granulation tissue, hyalinization, fibrosis, and inflammation. Secondly, viable tumor and the other tumor components must be localized in relation to anatomical landmarks as this is a prerequisite for local resection. Finally, imaging studies can be used to determine the effect of neo-adjuvant therapy. Although it has been claimed that establishing the effect of neo-adjuvant therapy can be done without comparing follow-up imaging with initial pre-treatment imaging, accuracy of this assessment seems to benefit from availability of pre- and post- treatment imaging studies [5].

Currently, however, the effect of neo-adjuvant therapy is established on histopathological analysis of the resected specimen [6]. Histologic good response to neoadjuvant treatment has been shown to be prognostic for survival [7]. Research is focused on identifying imaging parameters that reflect this histopathological response, and that thus may be used as biomarkers to select patients for potentially successful (quality of life and overall survival) neo-adjuvant treatment, and to determine the effect of this treatment prior to termination of the treatment protocol allowing personalized treatment. Until recently, the only accepted indication of imaging in guiding (neo)-adjuvant treatment was the use of PET-CT in GIST [8].

1.2 Imaging modalities and algorithm:

1.2.1 Timepoint to assess the effect of neo-adjuvant therapy:

- Unless there is clinical suspicion on tumor progression, imaging to assess the effect of neo-adjuvant therapy should be done after termination of neoadjuvant therapy and as close as possible to the moment of resection. Especially when radiotherapy has been used, imaging should be done 4-6 weeks after termination of radiotherapy.

Up to 6 weeks following termination of radiotherapy marked edematous and inflammatory changes adversely affect interpretation of imaging. Whenever possible imaging should therefore be scheduled after this period [9] .

1.2.2. Type of imaging:

- As the goal of the various types of neo-adjuvant therapy is reduction of viable tumor tissue, the type of imaging required is not dependent on the type of neo-adjuvant therapy given. Multiparametric imaging combining MRI* (angiogenesis, perfusion, permeability, cell density) and 18-FDG-PET-CT (glucose metabolism) can be used to detect viable tumor and therapy induced changes based on a combination of morphologic and functional imaging.
- There is no role for radiography, Tc99m bone scan, or image guided biopsy in monitoring the effect of neo-adjuvant therapy.
- The analysis of functional imaging parameters is moving from the use of descriptive semantic features (vascularity, cell density, glucose metabolism, hypoxia, Ph) to radiomics which uses high dimensional semantic and agnostic (quantification of voxel, intervoxel, or pattern values) data.

---

* (MRI in general provides the best soft tissue contrast and serves to exactly assess the anatomic structures)

The aim of neo-adjuvant therapy is improvement of oncological outcome by reduction of viable tumor in the primary site, and, depending on the tumor type and in the case of systemic neo-adjuvant therapy, also ablation of already detected or occult metastatic disease. For metastatic disease see question B, this part focuses on primary tumor assessment.

Multiparametric imaging with 18-FDG-PET-CT, Doppler US, dynamic contrast enhanced MRI, and diffusion weighted MRI can be used to assess the effect of neo-adjuvant therapy, but the parameters that are valuable per tumor type, and its accuracy have yet to be determined and validated [4]. There are exceptions in which morphology, in particular reduction in tumor volume and fatty maturation of myxoid liposarcoma, correlates with histopathologic classification of response [10, 11] .

Ultimately successful validation of imaging parameters may change the execution of treatment protocols (selection of drugs, dose modulation in radiotherapy, continuing or aborting), and even the selection of patients who will benefit of specific treatment protocols when response can be detected before starting therapy [4, 12].

Regarding tumor vascularity the imaging targets are vascular density and perfusion (DCE-MRI perfusion parameters of time intensity curves, Doppler US), permeability and vascular resistance of tumor vessels (Doppler US resistive index, shunting, DCE-MRI permeability parameters) [5, 13-15]. Because of angiogenesis and lack of muscle in the wall of tumor vessels in association with larger spaces between endothelial cells, perfusion and permeability are higher in viable tumor [16] .

Although US is not an accurate technique to visualize components of residual viable tumor within the entire tumor mass, Doppler US and contrast enhanced US have been successfully used to evaluate the response to therapy. However, US has not been incorporated in clinical practice [13, 17].

Analysis of DCE-MRI can be done qualitatively (shape of time intensity curves), semi-quantatively (quantifying the shape of the time intensity curve, including the time interval between start of arterial and lesional enhancement. An interval $\leq\ 6\,sec\,\text{is consistent with viable tumor}$), and quantitively (Tofts two compartment model) [14]. When T1-mapping is included in the protocol, the permeability parameters defined in the two-compartment model of Tofts can be calculated (volume transfer constant (Ktrans), the fractional volume of the extravascular-extracellular space (v_e), the rate constant (k_{ep}, where $k_{ep} = k^{trans}/v_e$), and the fractional volume of the plasma space (v_p) [14]. The motion of small Gd-chelate contrast agents between vessels and interstitium serve as a biomarker in this model and are function of capillary permeability, total vascular cross-sectional area, interstitial pressure, volume of extracellular space, contrast agent injection rate, and cardiac output. The values in this quantitative method depend, however, heavily on acquisition and post-processing methodology. Especially the arterial input function of variable, as this can be measured, or assumed based on statistical methods, causing variability in data [18].

One of the features of malignancy is uncontrolled growth leading to high cell density. Cell density relative to interstitial space can be assessed with DWI-MRI, and DW kurtuosis MRI. Cell density is higher and diffusion lower in viable neoplasm relative to that of normal tissue and reactive changes, but can be low in therapy induced changes such as necrosis [19-21]. Because of the dependency of ADC values on patient characteristic, acquisition and post-processing methods, it is difficult to define cut-off values. However, minimal ADC values of > 2 mm^2/sec (sensitivity 100%, specificity 61.1%) and average ADC values of > 2.2 mm^2/sec (sensitivity 50%, specificity 77.8%) have been reported as useful cut-off value consistent with good response [5]. Recently, whole-tumor texture analysis of multisequence MRI imaging, including ADC maps, T2w, and contrast enhanced T1w sequences proved superior to Recist 1.1. and AJCC staging, in predicting response to neoadjuvant radiotherapy and targeted therapy in patients with soft tissue sarcoma [22].

Glucose metabolism is deregulated and higher in high grade neoplasm, and glucose analogue imaging with 18-FDG-PET has been reported to show decrease or even shut down of tumor metabolism after successful neo-adjuvant therapy. A larger decrease of SUV_{max} in responders as opposed to nonresponders has a high sensitivity, but low specificity [23].

Radiomics describes quantitative image feature extraction and (texture) analysis. From voxels generated by CT or MRI, statistical and histogram parameters analyzed, include mean intensity, standard deviation, entropy, mean of positive pixels, kurtosis, and skewness. These agnostic parameters can be analyzed with artificial intelligence algorithms and can be used to quantify response to neo-adjuvant treatment [4, 20, 24-27].

Assessing the response to neoadjuvant treatment is based on the amount of viable tumor relative to other tumor components (necrosis, hemorrhage, inflammation, fibrosis, hyalinization, granulation tissue). The variability in sarcoma types, and correlation between biomarkers and clinical outcome in these different types is complicated. However, there is a classification system defined by the EORTC-STBSG group, which recognizes 5 classifications from A (very good) to E poor. In category A there are no stainable tumor cells. In category B this number is <1%, and in category C this is >1 till 10% [6] .

To correlate with the histopathological system the same categories should be used in imaging. However, as we currently have no validated accuracies of the various functional imaging parameters, a cut off value of 5% has been used to differentiate good from poor response [5, 28] . Change in tumor volume is, with some exceptions, not considered to be a reliable biomarker for response [15, 29-31] . Especially in radiotherapy increase in size is common (31%) and is not related to poor outcome [30, 32]. RECIST 1.1 criteria, which are based on volume, do not correlate with clinical outcome [33-35]. When enhancement features documented with CT (CHOI), or MRI (modified Choi criteria) are added the accuracy increases [35]. Models including texture analysis are promising [22].

1.3 Imaging parameters and report:

1.3.1 MRI:

- The MR protocol consists of morphologic and functional components. The morphologic part of the acquisition protocol is the same as the initial diagnostic MRI protocol (Table 1). The functional part, consisting of dynamic contrast enhanced MRI and diffusion weighted MRI, needs to be done not only in the follow-up protocol, but also in the initial diagnostic protocol, as changes in functional parameters facilitate response assessment.
- For MRI scans performed during and after neoadjuvant treatment, the same findings need to be described in the report as at baseline (*see [36]* .
- For MRI scans performed during and after neoadjuvant treatment, additionally, after neoadjuvant therapy specific findings need to be mentioned regarding treatment response and re-evaluation of resectability. Specifically, this regards location and size of viable residual tumor, changes in tumor volume and signal intensities, enhancement and diffusion characteristics.
- Machine learning approaches may become applicable for segmentation and evaluation of treatment response in STS.

Parameters:

The morphologic part of the acquisition protocol should be performed as described for the the initial diagnostic MRI protocol [36, 37] *also see section 1*).

For the functional part dynamic contrast enhanced MRI should be performed with a temporal resolution of at least 3 sec, and should include T1 mapping. Image acquisition should start directly before starting an IV bolus injection of Gd-chelate with a concentration of 0.2 mL/kg at an injection rate of 4 mL/s. Diffusion MRI should be done with a fast acquisition protocol using at least two b values (typically 0 -50 and 800-1000 s/mm^2) [18]. Although diffusion weighted MRI is a quantitative method, the ADC values dependent on many factors including hardware used in data acquisition, pulse sequence design, field inhomogenity (for instance previous surgery), and post processing software [38, 39] It is therefore recommended that initial pre-treatment and follow-up MRI is done

with the same protocols using identical acquisition and post-processing hard- and software. Variation in ADC values may be up to 16% [38, 40]

FDG-PET-CT should be done according to the EANM protocol version 2.0 [41].

<u>Report:</u>

1. Tumor size: diameters and volume of the tumor. Maximum diameters should be measured similar to the baseline scan and given in three dimensions. Volume measurements may be performed and compared to baseline (automatically in PACS or with dedicated postprocessing software).
2. Signal intensity changes on native MR sequences: on T1 (e.g. hemorrhage, fatty conversion in myxoid liposarcoma post radiotherapy) and T2 (e.g. increased T2 signal in keeping with necrosis, decreased T2 signal in keeping with hyalinization/fibrotic tissue).
3. Pattern of enhancement and enhancing fraction should be described based on the static post contrast images.
4. Perfusion: qualitative (type of Time Intensity Curve (TIC)), semi-quantitative (time interval between start arterial and lesionsl enhancement, wash in rate, time to peak, iAUC60), or quantitative (K trans, Kep, Ve, Vp) [27, 42]
5. Diffusion weighted imaging: qualitative (diffusion restriction present or not) or quantitative ADC value (minimum, mean, median, (10th, 25th, 75th, 90th percentiles, skew and kurtosis) [43]
6. Peritumoral tails: presence, extent and size (e.g. in myxoid fibrosarcoma)
7. Relation with and distance to the neurovascular bundle (measured on the axial T1), changes compared to baseline [11]
8. Satellite macroscopic lesions beyond the pseudo-capsule of the tumor.

In the future, machine learning approaches may become applicable for segmentation and evaluation of treatment response in STS [27, 44].

1.3.2 PET/CT:

- FDG-PET/CT should be done according to the latest EANM protocol version.

FDG-PET-CT should be performed according to the latest EANM protocol version (currently, version 2.0 [41].

1. *Casali PG, Abecassis N, Aro HT, et al. (2018) Soft tissue and visceral sarcomas: ESMO-EURACAN Clinical Practice Guidelines for diagnosis, treatment and follow-up. Ann Oncol* ***29****(Suppl 4):iv51-iv67*
2. *Gronchi A, Ferrari S, Quagliuolo V, et al. (2017) Histotype-tailored neoadjuvant chemotherapy versus standard chemotherapy in patients with high-risk soft-tissue sarcomas (ISG-STS 1001): an international, open-label, randomised, controlled, phase 3, multicentre trial. Lancet Oncol* ***18****(6):812-822*
3. *Benz MR, Czernin J, Allen-Auerbach MS, et al. (2009) FDG-PET/CT imaging predicts histopathologic treatment responses after the initial cycle of neoadjuvant chemotherapy in high-grade soft-tissue sarcomas. Clin Cancer Res* ***15****(8):2856-63*
4. *Kalisvaart GM, Bloem JL, Bovee J, et al. (2021) Personalising sarcoma care using quantitative multimodality imaging for response assessment. Clin Radiol* ***76****(4):313 e1-313 e13*
5. *Soldatos T, Ahlawat S, Montgomery E, Chalian M, Jacobs MA and Fayad LM (2016) Multiparametric Assessment of Treatment Response in High-Grade Soft-Tissue Sarcomas with Anatomic and Functional MR Imaging Sequences. Radiology* ***278****(3):831-40*
6. *Wardelmann E, Haas RL, Bovee JV, et al. (2016) Evaluation of response after neoadjuvant treatment in soft tissue sarcomas; the European Organization for Research and Treatment of Cancer-Soft Tissue and Bone Sarcoma Group (EORTC-STBSG) recommendations for pathological examination and reporting. Eur J Cancer* ***53****:84-95*
7. *Bonvalot S, Wunder J, Gronchi A, et al. (2021) Complete pathological response to neoadjuvant treatment is associated with better survival outcomes in patients with soft tissue sarcoma: Results of a retrospective multicenter study. Eur J Surg Oncol* ***47****(8):2166-2172*
8. *Farag S, Geus-Oei LF, van der Graaf WT, et al. (2018) Early Evaluation of Response Using (18)F-FDG PET Influences Management in Gastrointestinal Stromal Tumor Patients Treated with Neoadjuvant Imatinib. J Nucl Med* ***59****(2):194-196*
9. *Messiou C, Bonvalot S, Gronchi A, et al. (2016) Evaluation of response after pre-operative radiotherapy in soft tissue sarcomas; the European Organisation for Research and Treatment of Cancer-Soft Tissue and Bone Sarcoma Group (EORTC-STBSG) and Imaging Group recommendations for radiological examination and reporting with an emphasis on magnetic resonance imaging. Eur J Cancer* ***56****:37-44*
10. *Roberge D, Skamene T, Nahal A, Turcotte RE, Powell T and Freeman C (2010) Radiological and pathological response following pre-operative radiotherapy for soft-tissue sarcoma. Radiother Oncol* ***97****(3):404-7*
11. *Lansu J, Braam PM, van Werkhoven E, et al. (2021) A moderate dose of preoperative radiotherapy may improve resectability in myxoid liposarcoma. Eur J Surg Oncol* ***47****(10):2633-2639*
12. *Hong L, Alektiar KM, Hunt M, Venkatraman E and Leibel SA (2004) Intensity-modulated radiotherapy for soft tissue sarcoma of the thigh. Int J Radiat Oncol Biol Phys* ***59****(3):752-9*
13. *Lassau N, Paturel-Asselin C, Guinebretiere JM, et al. (1999) New hemodynamic approach to angiogenesis: color and pulsed Doppler ultrasonography. Invest Radiol* ***34****(3):194-8*
14. *Tofts PS (1997) Modeling tracer kinetics in dynamic Gd-DTPA MR imaging. J Magn Reson Imaging* ***7****(1):91-101*
15. *Shapeero LG, Vanel D, Verstraete KL and Bloem JL (1999) Dynamic Contrast-Enhanced MR Imaging for Soft Tissue Sarcomas. Semin Musculoskelet Radiol* ***3****(2):101-114*
16. *Alic L, van Vliet M, van Dijke CF, Eggermont AM, Veenland JF and Niessen WJ (2011) Heterogeneity in DCE-MRI parametric maps: a biomarker for treatment response? Phys Med Biol* ***56****(6):1601-16*
17. *Lassau N, Lamuraglia M, Vanel D, et al. (2005) Doppler US with perfusion software and contrast medium injection in the early evaluation of isolated limb perfusion of limb sarcomas: prospective study of 49 cases. Ann Oncol* ***16****(7):1054-60*
18. *Messiou C, Orton M, Ang JE, et al. (2012) Advanced solid tumors treated with cediranib: comparison of dynamic contrast-enhanced MR imaging and CT as markers of vascular activity. Radiology* ***265****(2):426-36*

19. *Winfield JM, Miah AB, Strauss D, et al. (2019) Utility of Multi-Parametric Quantitative Magnetic Resonance Imaging for Characterization and Radiotherapy Response Assessment in Soft-Tissue Sarcomas and Correlation With Histopathology. Front Oncol* ***9****:280*
20. *Ogawa M, Kan H, Arai N, et al. (2019) Differentiation between malignant and benign musculoskeletal tumors using diffusion kurtosis imaging. Skeletal Radiol* ***48****(2):285-292*
21. *Li SP and Padhani AR (2012) Tumor response assessments with diffusion and perfusion MRI. J Magn Reson Imaging* ***35****(4):745-63*
22. *Miao L, Cao Y, Zuo L, et al. (2023) Predicting pathological complete response of neoadjuvant radiotherapy and targeted therapy for soft tissue sarcoma by whole-tumor texture analysis of multisequence MRI imaging. Eur Radiol* ***33****(6):3984-3994*
23. *Benz MR, Tchekmedyian N, Eilber FC, Federman N, Czernin J and Tap WD (2009) Utilization of positron emission tomography in the management of patients with sarcoma. Curr Opin Oncol* ***21****(4):345-51*
24. *Esser M, Kloth C, Thaiss WM, et al. (2018) CT-response patterns and the role of CT-textural features in inoperable abdominal/retroperitoneal soft tissue sarcomas treated with trabectedin. Eur J Radiol* ***107****:175-182*
25. *Crombe A, Perier C, Kind M, et al. (2019) T(2) -based MRI Delta-radiomics improve response prediction in soft-tissue sarcomas treated by neoadjuvant chemotherapy. J Magn Reson Imaging* ***50****(2):497-510*
26. *Tian F, Hayano K, Kambadakone AR and Sahani DV (2015) Response assessment to neoadjuvant therapy in soft tissue sarcomas: using CT texture analysis in comparison to tumor size, density, and perfusion. Abdom Imaging* ***40****(6):1705-12*
27. *Spinnato P, Kind M, Le Loarer F, et al. (2022) Soft Tissue Sarcomas: The Role of Quantitative MRI in Treatment Response Evaluation. Acad Radiol* ***29****(7):1065-1084*
28. *Eilber FC, Rosen G, Eckardt J, et al. (2001) Treatment-induced pathologic necrosis: a predictor of local recurrence and survival in patients receiving neoadjuvant therapy for high-grade extremity soft tissue sarcomas. J Clin Oncol* ***19****(13):3203-9*
29. *Delisca GO, Alamanda VK, Archer KR, Song Y, Schwartz HS and Holt GE (2013) Tumor size increase following preoperative radiation of soft tissue sarcomas does not affect prognosis. J Surg Oncol* ***107****(7):723-7*
30. *Canter RJ, Martinez SR, Tamurian RM, et al. (2010) Radiographic and histologic response to neoadjuvant radiotherapy in patients with soft tissue sarcoma. Ann Surg Oncol* ***17****(10):2578-84*
31. *Shapeero LG, Vanel D, Verstraete KL and Bloem JL (2002) Fast magnetic resonance imaging with contrast for soft tissue sarcoma viability. Clin Orthop Relat Res (397):212-27*
32. *Miki Y, Ngan S, Clark JC, Akiyama T and Choong PF (2010) The significance of size change of soft tissue sarcoma during preoperative radiotherapy. Eur J Surg Oncol* ***36****(7):678-83*
33. *le Grange F, Cassoni AM and Seddon BM (2014) Tumour volume changes following pre-operative radiotherapy in borderline resectable limb and trunk soft tissue sarcoma. Eur J Surg Oncol* ***40****(4):394-401*
34. *Look Hong NJ, Hornicek FJ, Harmon DC, et al. (2013) Neoadjuvant chemoradiotherapy for patients with high-risk extremity and truncal sarcomas: a 10-year single institution retrospective study. Eur J Cancer* ***49****(4):875-83*
35. *Stacchiotti S, Collini P, Messina A, et al. (2009) High-grade soft-tissue sarcomas: tumor response assessment--pilot study to assess the correlation between radiologic and pathologic response by using RECIST and Choi criteria. Radiology* ***251****(2):447-56*
36. *Noebauer-Huhmann IM, Vanhoenacker FM, Vilanova JC, et al. (2023) Soft tissue tumor imaging in adults: European Society of Musculoskeletal Radiology-Guidelines 2023-overview, and primary local imaging: how and where? Eur Radiol*
37. *Huijgen WHF, van Rijswijk CSP and Bloem JL (2019) Is fat suppression in T1 and T2 FSE with mDixon superior to the frequency selection-based SPAIR technique in musculoskeletal tumor imaging? Skeletal Radiology* ***48****(12):1905-1914*

38. *Donati OF, Chong D, Nanz D, et al. (2014) Diffusion-weighted MR imaging of upper abdominal organs: field strength and intervendor variability of apparent diffusion coefficients. Radiology* ***270****(2):454-63*
39. *Koh DM, Collins DJ and Orton MR (2011) Intravoxel incoherent motion in body diffusion-weighted MRI: reality and challenges. AJR Am J Roentgenol* ***196****(6):1351-61*
40. *Michoux NF, Ceranka JW, Vandemeulebroucke J, et al. (2021) Repeatability and reproducibility of ADC measurements: a prospective multicenter whole-body-MRI study. Eur Radiol* ***31****(7):4514-4527*
41. *Boellaard R, Delgado-Bolton R, Oyen WJ, et al. (2015) FDG PET/CT: EANM procedure guidelines for tumour imaging: version 2.0. Eur J Nucl Med Mol Imaging* ***42****(2):328-54*
42. *Drape JL (2013) Advances in magnetic resonance imaging of musculoskeletal tumours. Orthop Traumatol Surg Res* ***99****(1 Suppl):S115-23*
43. *Ahlawat S, Khandheria P, Del Grande F, et al. (2016) Interobserver variability of selective region-of-interest measurement protocols for quantitative diffusion weighted imaging in soft tissue masses: Comparison with whole tumor volume measurements. J Magn Reson Imaging* ***43****(2):446-54*
44. *Blackledge MD, Winfield JM, Miah A, et al. (2019) Supervised Machine-Learning Enables Segmentation and Evaluation of Heterogeneous Post-treatment Changes in Multi-Parametric MRI of Soft-Tissue Sarcoma. Front Oncol* ***9****:941*

Section 2. Post-therapeutic surveillance in soft tissue sarcoma*

*Exclusions: GIST, uterine sarcomas, soft tissue metastases from non-musculoskeletal primaries

2.1 Overall evidence

- Still, there are no evidence-based recommendations for routine follow-up in surgically treated sarcomas.

Comments:

With improved therapies and better prognosis, the role of surveillance is increasing. The development of evidence-based recommendations is hampered by the heterogeneity of soft tissue sarcoma entities. The current “one-follow-up-strategy-fits-all” approach may neglect the differing degrees of risk in the diverse soft tissue tumor population and result in excessive surveillance for some patients. Hence, the assessment of individual risk remains important [1]. Also, it has to be taken into account whether LR or metastases can be detected by the chosen method, and whether this detection is important for the prognosis of the patient [2]. Recently, models have been designed to assess the personalized risk and tailor follow up based on some risk factors of local recurrence, amongst which gender, size, histology, neo- and adjuvant radiotherapy (RTX), and margins provided relevant information [3]. Depending on the entity, adjusting the time intervals and the overall surveillance period may be needed [4].

Another important factor is the location of the tumor. Recurrences are more common in sarcomas of the trunk [5] with almost 40% of cases after 5 years, compared with about 20% in extremity sarcomas [6]. In general, localizations in the deep retroperitoneal and head and neck are associated with higher recurrence rates[7, 8]. LR is also observed more often on the upper extremity than on the lower extremity [9].

Radiotherapy further decreases local recurrence [3, 10]. With limb-preserving resection and postoperative radiation, rates of 9% after 5 years and 12% after 10 years can be achieved [11, 12].

2.2 Timeline

2.2.1: Follow-up intervals

We would generally advocate:

- baseline follow-up no earlier than 3m post treatment*
- In high grade sarcoma: Year 1-3 every 3-4 months, year 4-5 every 6 months, year 6-10 annually*
- In low grade sarcoma: Year 1-3 every 6 months, year 4-10 annually°
- In grade 1 sarcoma with initial R0 resection^, patient-initiated follow up, instead of regular intervals, may be considered after year 5 in compliant patients.

*(after resection or adjuvant therapy, whatever comes latest)

°For modified strategies (intervals and modalities) in special entities and conditions please see under "Individualized strategy "

^R0 resection indicates a microscopically margin-negative resection, in which no gross or microscopic tumor remains in the primary tumor bed.

Comments:

Baseline local surveillance:

First local surveillance imaging should be scheduled no earlier than 3 m after treatment (surgery+/- radiotherapy) to minimize changes from early post treatment changes (edema, collections, etc.) [13]

Local recurrence (LR):

In high grade sarcomas, the LR rate is higher than in low grade sarcomas [3, 5, 6, 14, 15] .

Most early recurrences are observed in high-grade sarcomas within the first 2 to 3 years of surveillance [14]. After combined surgery and Rtx, more than 90% of first local recurrencies are observed within the first 5y, and all recurrencies within 15y [7]. A positive microscopic resection margin is another adverse prognostic factor for local control [16]. Large (>10cm) sarcomas are associated with late (> 5 years) LR, with recommendation of long-term follow-up [17].

Influence of low tumor grade:

Recurrence seems unlikely in low grade sarcomas after R0 resection [18]. Low grade tumors re-occur at a constant rate throughout follow-up [14]. While late recurrence is less frequent it may occur in low-grade STS and may manifest with higher grade[19, 20].

LHowever late (> 5 years) first LR is very rare in grade I tumors [17]. Thus, long-term follow-up by imaging can be regarded unnecessary in those patients [17].

Influence of entities and tumor location:

Well-differentiated liposarcoma, myxoid liposarcoma and leiomyosarcoma are associated with late (> 5 years) LR [17, 21]. Retroperitoneal sarcomas and intra-abdominal lesions have a propensity for early LR [22]; however retroperitoneal sarcomas are also associated with late (> 5 years) LR [17, 21]. Thus, long-term follow-up to detect late local disease recurrence can be recommended in patients with retroperitoneal sarcomas [17].

Metastatic/distant recurrence:

Factors that are associated with metastatic recurrence are high tumor grade and tumor size >5 cm [7]. In high grade sarcomas, the rate of distant metastases is high in the first two years and decreases afterwards [14]. FNCLCC high-grade sarcoma is associated with late (> 5 years) metastatic recurrences [17]. Entities for which a higher rate of metastatic recurrence has been described are leiomyosarcoma, rhabdomyosarcoma, synovial sarcoma, epithelioid sarcoma [7], undifferentiated sarcoma and de-differentiated liposarcoma [23]. In retroperitoneal sarcomas, the entities high-grade leiomyosarcoma, solitary fibrous tumor and high-grade liposarcoma are associated with an increased cumulative incidence of distant recurrence [21]. Low grade sarcomas rarely metastasize [14].
Repeat resections of recurrent pulmonary metastasis show a significantly better prognosis than those with only one resection [24].

2.2.2: Endpoint

- Regular follow-up should be carried out during the first 10y after the initial diagnosis.
- Regular annual follow-up should be continued longer than 10y in patients with well-differentiated (retroperitoneal) liposarcomas and myxoid liposarcoma.
- In case of recurrence, the surveillance algorithm should restart.

Comments:

Few LR or metastases are found at follow-up beyond 10 years [14]
Well-differentiated liposarcoma, myxoid liposarcoma, *and s*ynovial sarcoma are associated with late LR as long as 15 years from diagnosis [21, 25].
For comparison with other guidelines, please see Supplementary Table S1.

2.3 Modalities

2.3.1: Role of imaging

- The inclusion of Imaging in follow-up is necessary, especially in high grade STS.
- The ability to offer successful salvage treatment of recurrent disease supports systematic imaging surveillance and early detection of recurrence*.
- A fixed follow-up schedule for patients with STS permits timely detection of LR and metastatic disease.

* For exceptions depending on sarcoma subtype, and modifying factors such as patient condition, please see below

---

Comments:

Apart from clinical and laboratory examinations, follow-up should include imaging for local recurrence and metastatic disease. There is debate about whether imaging should be performed regularly or only in symptomatic cases [2, 26-29].

In a study including soft tissue sarcomas in the extremities and trunk wall, local imaging (mainly MRI) identified a statistically significant larger amount of LR than clinical examination did [30]. This is in accordance with a study, where surveillance by MRI detected a significant number of clinically undetectable LRs (11% (34/325)), especially for LRs in the thigh or buttock, small LRs or LRs without mass formation [31]. In another study, about one third of LRs were detected by routine imaging only [32], however without factors (such as patient, tumor, or therapeutic characteristics) that could define a subgroup of patients that more or less likely benefit from surveillance by imaging [32]. Another study observed that 46 of 87 patients with LR of a soft tissue sarcoma could have been diagnosed earlier with routine cross sectional imaging (only one of those 46 with pelvic sarcoma) [33].

In <u>deep sarcomas</u>, LR are often clinically undetectable [23].

In <u>extremity STS</u> post surgery and RTx, 1 of 11 local recurrences in a study of 114 patients was clinically undetectable and only revealed by MRI. [34] In another study of 124 patients, 2 of 11 local recurrences of limb sarcoma were only seen by MRI; both after R1 resection, while the authors also observed false positive cases [35]. The knowledge of surgical margins substantially increases the value of MRI in detecting recurrent soft-tissue sarcoma [36].

For various reasons, especially after RTX, there may be marked scar tissue formation [37], hampering the palpation of LR even more.

Influence on prognosis:

The median delay between initial surgery and detection of LR was shorter when LR was identified by imaging (median: 20.1 months [min ¼ 5.3-max ¼ 35.7]) than by clinical examination (median: 28.6 Months; range 2.0- 52.4) [23].

In clinically undetectable LRs, patients with MRI-detected LR showed a (non-significant) trend toward a better survival [31].

In some of the patients, in whom LR is detected earlier by imaging, the extent of surgery can be decreased, amputation can be avoided; or (if applicable) the radiation field can be decreased [33].

2.3.2. Imaging modalities in general

- MRI is the method of choice for local and loco-regional surveillance of soft tissue sarcomas
- In sarcomas of the mediastinum, retroperitoneum and visceral sites, CT may be indicated instead of MRI for local and loco-regional surveillance.
- In limb sarcomas, US represents a valuable alternative for the assessment of local recurrence if MRI is inconclusive due to artifacts, in cases where MRI is contraindicated, or in rare cases where MRI is not available.
- In subcutaneous low-grade lesions, and given that potential LR would be likely palpable, local surveillance with ultrasound may be considered instead of MRI.
- For metastatic disease, chest CT should be performed (For modified strategies in special entities and conditions please see under “Individualized strategy “)
- FDG-PET/CT can be a useful problem-solving tool if another study is equivocal

---

Comments:

Local and loco-regional surveillance:

MRI is the method of choice for local and loco-regional follow-up [31, 32, 38-40].

In sarcomas of the mediastinum, retroperitoneum and visceral sites, if artifacts are anticipated in the study area, or if there are reasons not to undergo MRI, CT or PET/CT may be indicated instead of MRI for local and loco-regional surveillance [41-43]

In limb sarcomas, US (by an experienced sonographer) seems to be a cost-effective primary imaging alternative for exclusion of local recurrence [44], particularly in the presence of metallic hardware [45]. However, especially in the early postoperative period, close comparison with a baseline MRI is needed [46].

In case of large metallic hardware, dual-energy CT or CT using modern iterative reconstruction algorithms of raw datasets, or PET/CT can be considered as alternative or additive to MRI [47]. In MRI, the use of lower field strength and specific artifact suppression techniques can help to reduce metal artifacts [48, 49].

Whole-body surveillance

For pulmonary metastasis, recommended chest imaging modalities vary from chest X-ray [50] to chest X-ray or chest CT [27, 51], to chest CT alone [2, 52]. Chest CT proved to be superior in the detection of pulmonary metastases, compared to chest x-ray [30]. While some authors did not find survival benefit by the use of chest CT [53], another study observed a longer median survival after relapse if the diagnosis of metastatic relapse was made on planned chest-CT scan rather than chest X-ray [54].

FDG-PET/CT whole body can be a useful problem-solving tool if another study is equivocal, particularly in cases of suboptimal MRI, because of extensive metal artifacts, or where MRI is contra-indicated [32, 55, 56].

Most soft tissue sarcomas, especially the more aggressive ones, are metabolically active in FDG PET/CT [57]. In the latest NCCN Guidelines, the use of CT or PET/CT in sarcomas with propensity for lymph node metastases is recommended [27, 58] . In the future, larger data set evaluations with subsequent individualized risk assessment for sarcoma patients are expected to lead to adapted surveillance strategies, including refinement of indication for PET/CT. An increasing availability of PET/CT scanners, the development of novel tracers, as well as entity-based tracer avidity cutoff values may lead to broader implementation of the method.

2.3.3. Imaging parameters

Local MRI:

- The FOV should cover the whole surgical/post-therapeutic region.
- One anatomic landmark should be visible.
- Same sequence parameters as in primary diagnosis can be used, except for sites where modifications are required to reduce artifacts from metallic hardware.
- If possible/not contraindicated, contrast agent should be used.

Whole body MRI:

- For surveillance, the parameters of primary staging can be used.

Comments:

For surveillance of soft tissue sarcoma, in general, the US and MR techniques that have been used for primary imaging can also facilitate comparison of the examinations.
Color Doppler may help differentiate recurrent tumor mass from fibrous tissue or other non-vascularized tissue (hematoma, seroma) in the postoperative site [46], however, the lack of Doppler signal does not exclude recurrence [44].
In case of metallic hardware, lower MRI field strengths are preferred, and dedicated sequences that are optimized for minimizing susceptibility artifacts should be used [48, 49]. Although diffusion-weighted imaging is currently hampered by limited image quality, it facilitates the detection of recurrent lesions and, when evaluated in conjunction with other sequences, may increase confidence in diagnosing recurrence [59].

If possible, CE MRI should be used [60]. Contrast enhanced (CE) MRI also increases confidence in less experienced readers [61]. Dynamic contrast-enhanced MRI is useful in the differentiation of recurrent soft-tissue sarcoma and post-therapeutic alterations [62] such as fibrosis [36]. Radiomics is useful for the detection of local recurrence of STS, and for lesion characterization [63].

2.4. Individualized follow-up strategy

2.4.1. Myxoid liposarcoma (MLS)

- For the detection of metastases, WB-MRI is recommended (for local surveillance, additional dedicated local MRI is recommended).
- For the detection of metastases, in year 0-2, chest CT is recommended every 3 m, followed by chest radiographs every 6m up to year 5 thereafter.

Comments:

Because of the unconventional metastatic behavior of myxoid liposarcoma (MLS), with recurrence sites that differ from other soft tissue sarcomas (with a high proportion of extrapulmonary metastases and low incidence of pulmonary metastases), and because of its low PET-avidity [64-66] WBMRI has been recommended for staging and follow-up [67-70]. A possible protocol contains at least coronal and axial STIR and a coronal T1w sequence [68].

In future, the surveillance intervals and modalities may be adjusted, dependent on the likelihood for LR and metastases (which seem to depend on resection margins, age, grading and the round cell content of the tumor) [66, 67, 71].

A potential surveillance protocol could include a dedicated MRI of the primary site, whole body MRI and chest CT. The time intervals may follow those described for sarcoma in general (depending on the tumor grade; surveillance of sarcoma with bad response to neo-adjuvant RTX is regarded equivalent to high grade MLS) [67, 68].

2.4.2. Other entities which require specific follow-up imaging strategies

---

- Alveolar soft part sarcoma, Angiosarcoma, epithelioid sarcoma, clear cell sarcoma, rhabdomyosarcoma, leiomyosarcoma, undifferentiated pleomorphic sarcoma, retroperitoneal (well-/) dedifferentiated liposarcoma

---

Comments:

Metastatic spread of soft tissue sarcomas mainly is hematogenous, and pulmonary metastases are most common, accounting for about 75 to 80% of metastases. Other sites are less commonly involved.

Osseous metastases occur in about 10% of soft tissue sarcoma patients [72]. Higher incidences have been described for alveolar soft part sarcomas [73, 74], angiosarcomas [73], leiomyosarcomas [75], undifferentiated pleomorphic sarcomas [72], and dedifferentiated liposarcomas [73]. Eighty percent of those osseous metastases are lytic [72], and fluorodeoxyglucose (FDG)-PET may be useful in these tumors.

Lymph node involvement is rare (about 3% of sarcomas) and is more frequent in high-grade rhabdomyosarcoma, clear cell sarcomas, epithelioid sarcoma, angiosarcoma, and synovial sarcoma [76, 77].

In epithelioid sarcoma [78], angiosarcoma [79, 80], leiomyosarcoma[80, 81], solitary fibrous tumor (SFT) and PEComa [80], abdominal imaging should be considered according to the literature.

The literature assessing the value of (FDG)-PET for surveillance of soft tissue sarcomas is limited. As soft tissue sarcomas are rare, the articles do not differentiate between entities [47, 55, 56, 82]. However, it is known that high grade sarcomas correlate with high SUV max [56], and that recurrences tend to be higher grade, compared with the primary tumor [83]. False negative results were seen in entities such as well-differentiated liposarcoma and low-grade synovial sarcoma [84] or small size (inguinal lymph node metastases or tumor) [47, 55]. Of note, patient management was

changed in 72% of (correctly referred) patients after detection of LR and/or distant metastases by PET/CT [85].
In alveolar soft part sarcoma with lung metastases [86, 87], in clear cell sarcoma, and angiosarcoma[79], brain imaging should be considered [88].

2.5. Other points that should be considered in follow-up of soft tissue tumors

---

- Follow-up imaging should always be compared with previous images (especially those of the primary tumor, the baseline post-therapeutic images, as well as the most recent previous study). The study should ideally be performed on the same scanner, and the previous examination should be available (for copying sequence planes and parameters) and for comparative reading.
- Reports should contain the parameters that have been described for primary imaging (see there).
- Patients should be included by adequate information and encouragement to participate in the surveillance process.

Comments:

The follow-up MRI should be compared with the preoperative MRI (for morphology, site, and extent of the lesion), the baseline post-therapeutic one and the most recent one, at least.
Patients should be instructed carefully, and the patient's participation should be sought. This is in accordance with a study, where the vast majority felt that is was important to be included in decision-making about their follow-up regime [89]. In the same study, patients also preferred to remain in an expert sarcoma center rather than general practice for follow-up. In general, a 6-month interval seems to meet patient preferences for follow-up [89]. This is consistent with the personal experience of the expert panel regarding the importance of patient interaction: In our experience, an adequately informed, responsible patient is more compliant and confident, and less prone to loss for follow-up.

1. *Greenberg DD and Crawford B (2016) Surveillance Strategies for Sarcoma: Results of a Survey of Members of the Musculoskeletal Tumor Society. Sarcoma* ***2016****:8289509*
2. *Roberts CC, Kransdorf MJ, Beaman FD, et al. (2016) ACR Appropriateness Criteria Follow-Up of Malignant or Aggressive Musculoskeletal Tumors. J Am Coll Radiol* ***13****(4):389-400*
3. *Smolle MA, Sande MV, Callegaro D, et al. (2019) Individualizing Follow-Up Strategies in High-Grade Soft Tissue Sarcoma with Flexible Parametric Competing Risk Regression Models. Cancers (Basel)* ***12****(1)*
4. *Yahya Zaidi M and Cardona K (2018) Post-operative surveillance in soft tissue sarcoma: using tumor-specific recurrence patterns to direct approach. Chin Clin Oncol* ***7****(4):45*
5. *Sekimizu M, Ogura K, Yasunaga H, et al. (2019) Development of nomograms for prognostication of patients with primary soft tissue sarcomas of the trunk and extremity: report from the Bone and Soft Tissue Tumor Registry in Japan. BMC Cancer* ***19****(1):657*
6. *Italiano A, Le Cesne A, Mendiboure J, et al. (2014) Prognostic factors and impact of adjuvant treatments on local and metastatic relapse of soft-tissue sarcoma patients in the competing risks setting. Cancer* ***120****(21):3361-9*
7. *Zagars GK, Ballo MT, Pisters PW, Pollock RE, Patel SR and Benjamin RS (2003) Prognostic factors for disease-specific survival after first relapse of soft-tissue sarcoma: analysis of 402 patients with disease relapse after initial conservative surgery and radiotherapy. Int J Radiat Oncol Biol Phys* ***57****(3):739-47*
8. *Gronchi A, Strauss DC, Miceli R, et al. (2016) Variability in Patterns of Recurrence After Resection of Primary Retroperitoneal Sarcoma (RPS): A Report on 1007 Patients From the Multi-institutional Collaborative RPS Working Group. Ann Surg* ***263****(5):1002-9*
9. *Gerrand CH, Bell RS, Wunder JS, et al. (2003) The influence of anatomic location on outcome in patients with soft tissue sarcoma of the extremity. Cancer* ***97****(2):485-92*
10. *Smith HG, Memos N, Thomas JM, Smith MJ, Strauss DC and Hayes AJ (2016) Patterns of disease relapse in primary extremity soft-tissue sarcoma. Br J Surg* ***103****(11):1487-96*
11. *Novais EN, Demiralp B, Alderete J, Larson MC, Rose PS and Sim FH (2010) Do surgical margin and local recurrence influence survival in soft tissue sarcomas? Clin Orthop Relat Res* ***468****(11):3003-11*
12. *Felderhof JM, Creutzberg CL, Putter H, et al. (2013) Long-term clinical outcome of patients with soft tissue sarcomas treated with limb-sparing surgery and postoperative radiotherapy. Acta Oncol* ***52****(4):745-52*
13. *Bloem JL, Vriens D, Krol ADG, et al. (2020) Therapy-Related Imaging Findings in Patients with Sarcoma. Semin Musculoskelet Radiol* ***24****(6):676-691*
14. *Sawamura C, Matsumoto S, Shimoji T, Okawa A and Ae K (2014) How long should we follow patients with soft tissue sarcomas? Clin Orthop Relat Res* ***472****(3):842-8*
15. *Grobmyer SR and Brennan MF (2003) Predictive variables detailing the recurrence rate of soft tissue sarcomas. Curr Opin Oncol* ***15****(4):319-26*
16. *Potter BK, Hwang PF, Forsberg JA, et al. (2013) Impact of margin status and local recurrence on soft-tissue sarcoma outcomes. J Bone Joint Surg Am* ***95****(20):e151*
17. *Toulmonde M, Le Cesne A, Mendiboure J, et al. (2014) Long-term recurrence of soft tissue sarcomas: prognostic factors and implications for prolonged follow-up. Cancer* ***120****(19):3003-6*
18. *Watts AC, Teoh K, Evans T, Beggs I, Robb J and Porter D (2008) MRI surveillance after resection for primary musculoskeletal sarcoma. J Bone Joint Surg Br* ***90****(4):484-7*
19. *Grimer R, Judson I, Peake D and Seddon B (2010) Guidelines for the management of soft tissue sarcomas. Sarcoma* ***2010****:506182*
20. *Gibbs JF, Lee RJ, Driscoll DL, McGrath BE, Mindell ER and Kraybill WG (2000) Clinical importance of late recurrence in soft-tissue sarcomas. J Surg Oncol* ***73****(2):81-6*
21. *Tan MC, Brennan MF, Kuk D, et al. (2016) Histology-based Classification Predicts Pattern of Recurrence and Improves Risk Stratification in Primary Retroperitoneal Sarcoma. Ann Surg* ***263****(3):593-600*

22. *Brennan MF, Antonescu CR, Moraco N and Singer S (2014) Lessons learned from the study of 10,000 patients with soft tissue sarcoma. Ann Surg* ***260****(3):416-21; discussion 421-2*
23. *De Angelis F, Guy F, Bertaut A, et al. (2019) Limbs and trunk soft tissue sarcoma systematic local and remote monitoring by MRI and thoraco-abdomino-pelvic scanner: A single-centre retrospective study. Eur J Surg Oncol* ***45****(7):1274-1280*
24. *Liebl LS, Elson F, Quaas A, Gawad KA and Izbicki JR (2007) Value of repeat resection for survival in pulmonary metastases from soft tissue sarcoma. Anticancer Res* ***27****(4C):2897-902*
25. *Krieg AH, Hefti F, Speth BM, et al. (2011) Synovial sarcomas usually metastasize after >5 years: a multicenter retrospective analysis with minimum follow-up of 10 years for survivors. Ann Oncol* ***22****(2):458-67*
26. *von Mehren M, Randall RL, Benjamin RS, et al. (2018) Soft Tissue Sarcoma, Version 2.2018, NCCN Clinical Practice Guidelines in Oncology. J Natl Compr Canc Netw* ***16****(5):536-563*
27. *von Mehren M National Comprehensive Cancer Network. NCCN Clinical Practice Guidelines in Oncology: Soft Tissue Sarcoma. NCCN. Version Version 3.2023 — December 12, 2023 Available online: : https://www.nccn.org/professionals/physician_gls/pdf/sarcoma.pdf.*
28. *Expert Panel on Musculoskeletal I, Stanborough R, Demertzis JL, et al. (2022) ACR Appropriateness Criteria(R) Malignant or Aggressive Primary Musculoskeletal Tumor-Staging and Surveillance: 2022 Update. J Am Coll Radiol* ***19****(11S):S374-S389*
29. UK CR. Available from: https://www.cancerresearchuk.org/health-professional/cancer-statistics/statistics-by-cancer-type/soft-tissue-sarcoma.
30. *Hovgaard TB, Nymark T, Skov O and Petersen MM (2017) Follow-up after initial surgical treatment of soft tissue sarcomas in the extremities and trunk wall. Acta Oncol* ***56****(7):1004-1012*
31. *Park JW, Yoo HJ, Kim HS, et al. (2019) MRI surveillance for local recurrence in extremity soft tissue sarcoma. Eur J Surg Oncol* ***45****(2):268-274*
32. *England P, Hong Z, Rhea L, Hirbe A, McDonald D and Cipriano C (2020) Does Advanced Imaging Have a Role in Detecting Local Recurrence of Soft-tissue Sarcoma? Clin Orthop Relat Res* ***478****(12):2812-2820*
33. *George A, Grimer RJ and SL JJ (2018) Could Routine Magnetic Resonance Imaging Detect Local Recurrence of Musculoskeletal Sarcomas Earlier? A Cost-effectiveness Study. Indian J Orthop* ***52****(1):81-86*
34. *Cheney MD, Giraud C, Goldberg SI, et al. (2014) MRI surveillance following treatment of extremity soft tissue sarcoma. J Surg Oncol* ***109****(6):593-6*
35. *Labarre D, Aziza R, Filleron T, et al. (2009) Detection of local recurrences of limb soft tissue sarcomas: is magnetic resonance imaging (MRI) relevant? Eur J Radiol* ***72****(1):50-3*
36. *Hirschmann A, van Praag VM, Haas RL, van de Sande MAJ and Bloem JL (2020) Can we use MRI to detect clinically silent recurrent soft-tissue sarcoma? Eur Radiol* ***30****(9):4724-4733*
37. *Davis AM, O'Sullivan B, Turcotte R, et al. (2005) Late radiation morbidity following randomization to preoperative versus postoperative radiotherapy in extremity soft tissue sarcoma. Radiother Oncol* ***75****(1):48-53*
38. *Sedaghat S, Sedaghat M, Meschede J, Jansen O and Both M (2021) Diagnostic value of MRI for detecting recurrent soft-tissue sarcoma in a long-term analysis at a multidisciplinary sarcoma center. BMC Cancer* ***21****(1):398*
39. *Dammerer D, A VANB, Schneeweiss V and Schwabegger A (2020) Follow-up Strategies for Primary Extremity Soft-tissue Sarcoma in Adults: A Systematic Review of the Published Literature. In Vivo* ***34****(6):3057-3068*
40. *Verstraete KL and Lang P (2000) Bone and soft tissue tumors: the role of contrast agents for MR imaging. Eur J Radiol* ***34****(3):229-46*
41. *Ackman JB, Chung JH, Walker CM, et al. (2021) ACR Appropriateness Criteria® Imaging of Mediastinal Masses. Journal of the American College of Radiology* ***18****(5):S37-S51*
42. *Alvarez Alvarez R, Manzano A, Agra Pujol C, et al. (2023) Updated Review and Clinical Recommendations for the Diagnosis and Treatment of Patients with Retroperitoneal Sarcoma by the Spanish Sarcoma Research Group (GEIS). Cancers (Basel)* ***15****(12)*

43. Follmann M. *Adulte Weichgewebesarkome* 2024 [cited 2024; Available from: https://www.leitlinienprogramm-onkologie.de/leitlinien/adulte-weichgewebesarkome.
44. *Tagliafico A, Truini M, Spina B, et al. (2015) Follow-up of recurrences of limb soft tissue sarcomas in patients with localized disease: performance of ultrasound. Eur Radiol **25**(9):2764-70*
45. *Singer AD, Wong P, Umpierrez M, et al. (2020) The accuracy of a novel sonographic scanning and reporting protocol to survey for soft tissue sarcoma local recurrence. Skeletal Radiol **49**(12):2039-2049*
46. Gielen J, Vanhoenacker F, Ceulemans R, et al., *Ultrasound and Color Doppler Ultrasound of Soft Tissue Tumors and Tumorlike Lesions*, in *Imaging of Soft Tissue Tumors*. 2017, Springer-Verlag: Cham. p. 3-40.
47. *Park SY, Chung HW, Chae SY and Lee JS (2016) Comparison of MRI and PET-CT in detecting the loco-regional recurrence of soft tissue sarcomas during surveillance. Skeletal Radiol **45**(10):1375-84*
48. *Susa M, Oguro S, Kikuta K, et al. (2015) Novel MR imaging method--MAVRIC--for metal artifact suppression after joint replacement in musculoskeletal tumor patients. BMC Musculoskelet Disord **16**:377*
49. *Jungmann PM, Agten CA, Pfirrmann CW and Sutter R (2017) Advances in MRI around metal. J Magn Reson Imaging **46**(4):972-991*
50. *Dangoor A, Seddon B, Gerrand C, Grimer R, Whelan J and Judson I (2016) UK guidelines for the management of soft tissue sarcomas. Clin Sarcoma Res **6**:20*
51. *Gronchi A, Miah AB, Dei Tos AP, et al. (2021) Soft tissue and visceral sarcomas: ESMO-EURACAN-GENTURIS Clinical Practice Guidelines for diagnosis, treatment and follow-up(☆). Ann Oncol **32**(11):1348-1365*
52. *Patel SA, Royce TJ, Barysauskas CM, Thornton KA, Raut CP and Baldini EH (2017) Surveillance Imaging Patterns and Outcomes Following Radiation Therapy and Radical Resection for Localized Extremity and Trunk Soft Tissue Sarcoma. Ann Surg Oncol **24**(6):1588-1595*
53. *Gamboa AC, Ethun CG, Switchenko JM, et al. (2019) Lung Surveillance Strategy for High-Grade Soft Tissue Sarcomas: Chest X-Ray or CT Scan? J Am Coll Surg **229**(5):449-457*
54. *Blaye C, Kind M, Stoeckle E, et al. (2019) Local and Metastatic Relapse Features in Patients After a Primary Soft Tissue Sarcoma: Advocating for a Better-Tailored Follow-Up. Front Oncol **9**:559*
55. *Kassem TW, Abdelaziz O and Emad-Eldin S (2017) Diagnostic value of (18)F-FDG-PET/CT for the follow-up and restaging of soft tissue sarcomas in adults. Diagn Interv Imaging **98**(10):693-698*
56. *Macpherson RE, Pratap S, Tyrrell H, et al. (2018) Retrospective audit of 957 consecutive (18)F-FDG PET-CT scans compared to CT and MRI in 493 patients with different histological subtypes of bone and soft tissue sarcoma. Clin Sarcoma Res **8**:9*
57. *Fine GC, Covington MF, Koppula BR, et al. (2022) PET-CT in Clinical Adult Oncology-VI. Primary Cutaneous Cancer, Sarcomas and Neuroendocrine Tumors. Cancers (Basel) **14**(12)*
58. *Basile G, Mattei JC, Alshaygy I, et al. (2020) Curability of patients with lymph node metastases from extremity soft-tissue sarcoma. Cancer **126**(23):5098-5108*
59. *Zampa V, Aringhieri G, Tintori R, Rossi P, Andreani L and Franchi A (2023) The added value of the visual analysis of DWI in post-surgery follow-up of soft tissue sarcoma of the extremities: do we really need ADC? Radiol Med **128**(4):467-479*
60. *Afonso DP, Kosinski AS and Spritzer CE (2013) Following unenhanced MRI assessment for local recurrence after surgical resection of mesenchymal soft tissue tumors, do additional gadolinium-enhanced images change reader confidence or diagnosis? Eur J Radiol **82**(5):806-13*
61. *Chou SS, Hippe DS, Lee AY, et al. (2017) Gadolinium Contrast Enhancement Improves Confidence in Diagnosing Recurrent Soft Tissue Sarcoma by MRI. Acad Radiol **24**(5):615-622*

62. *Del Grande F, Subhawong T, Weber K, Aro M, Mugera C and Fayad LM (2014) Detection of soft-tissue sarcoma recurrence: added value of functional MR imaging techniques at 3.0 T. Radiology* ***271****(2):499-511*
63. *Tagliafico AS, Bignotti B, Rossi F, Valdora F and Martinoli C (2019) Local recurrence of soft tissue sarcoma: a radiomic analysis. Radiol Oncol* ***53****(3):300-306*
64. *Schwab JH, Boland PJ, Antonescu C, Bilsky MH and Healey JH (2007) Spinal metastases from myxoid liposarcoma warrant screening with magnetic resonance imaging. Cancer* ***110****(8):1815-22*
65. *Lin S, Gan Z, Han K, Yao Y and Min D (2015) Metastasis of myxoid liposarcoma to fat-bearing areas: A case report of unusual metastatic sites and a hypothesis. Oncol Lett* ***10****(4):2543-2546*
66. *Durr HR, Rauh J, Baur-Melnyk A, et al. (2018) Myxoid liposarcoma: local relapse and metastatic pattern in 43 patients. BMC Cancer* ***18****(1):304*
67. *Stevenson JD, Watson JJ, Cool P, et al. (2016) Whole-body magnetic resonance imaging in myxoid liposarcoma: A useful adjunct for the detection of extra-pulmonary metastatic disease. Eur J Surg Oncol* ***42****(4):574-80*
68. *Gorelik N, Reddy SMV, Turcotte RE, et al. (2018) Early detection of metastases using whole-body MRI for initial staging and routine follow-up of myxoid liposarcoma. Skeletal Radiol* ***47****(3):369-379*
69. *Seo SW, Kwon JW, Jang SW, Jang SP and Park YS (2011) Feasibility of whole-body MRI for detecting metastatic myxoid liposarcoma: a case series. Orthopedics* ***34****(11):e748-54*
70. *Paruthikunnan S, Gorelik N, Turcotte RE, Jung S, Alcindor T and Powell TI (2024) Clinical impact of whole-body MRI in staging and surveillance of patients with myxoid liposarcoma: a 14-year single-centre retrospective study. Eur Radiol*
71. *Haniball J, Sumathi VP, Kindblom LG, et al. (2011) Prognostic factors and metastatic patterns in primary myxoid/round-cell liposarcoma. Sarcoma* ***2011****:538085*
72. *Vincenzi B, Frezza AM, Schiavon G, et al. (2013) Bone metastases in soft tissue sarcoma: a survey of natural history, prognostic value and treatment options. Clin Sarcoma Res* ***3****(1):6*
73. *Yoshikawa H, Myoui A, Ochi T, et al. (1999) Bone Metastases from Soft Tissue Sarcomas. Semin Musculoskelet Radiol* ***3****(2):183-190*
74. *Sood S, Baheti AD, Shinagare AB, et al. (2014) Imaging features of primary and metastatic alveolar soft part sarcoma: single institute experience in 25 patients. Br J Radiol* ***87****(1036):20130719*
75. *Gordon RW, Tirumani SH, Kurra V, et al. (2014) MRI, MDCT features, and clinical outcome of extremity leiomyosarcomas: experience in 47 patients. Skeletal Radiol* ***43****(5):615-22*
76. *Mazeron JJ and Suit HD (1987) Lymph-Nodes as Sites of Metastases from Sarcomas of Soft-Tissue. Cancer* ***60****(8):1800-1808*
77. *Sherman KL, Kinnier CV, Farina DA, et al. (2014) Examination of national lymph node evaluation practices for adult extremity soft tissue sarcoma. J Surg Oncol* ***110****(6):682-8*
78. *Chase DR and Enzinger FM (1985) Epithelioid sarcoma. Diagnosis, prognostic indicators, and treatment. Am J Surg Pathol* ***9****(4):241-63*
79. *Meis-Kindblom JM and Kindblom LG (1998) Angiosarcoma of soft tissue: a study of 80 cases. Am J Surg Pathol* ***22****(6):683-97*
80. *Grimme FAB, Seesing MFJ, van Hillegersberg R, et al. (2019) Liver Resection for Hepatic Metastases from Soft Tissue Sarcoma: A Nationwide Study. Dig Surg* ***36****(6):479-486*
81. *Mankin HJ, Casas-Ganem J, Kim JI, Gebhardt MC, Hornicek FJ and Zeegen EN (2004) Leiomyosarcoma of somatic soft tissues. Clin Orthop Relat Res (421):225-31*
82. *Al-Ibraheem A, Buck AK, Benz MR, et al. (2013) 18F-Fluorodeoxyglucose positron emission tomography/computed tomography for the detection of recurrent bone and soft tissue sarcoma. Cancer* ***119****(6):1227-1234*
83. *Abatzoglou S, Turcotte RE, Adoubali A, Isler MH and Roberge D (2010) Local recurrence after initial multidisciplinary management of soft tissue sarcoma: is there a way out? Clin Orthop Relat Res* ***468****(11):3012-8*

84. *Shaban EAIN (2018) The usefulness of 18-F-FDG PET/CT for detection of adult soft tissue sarcomas local recurrence and distant metastases. Egyptian Journal of Radiology and Nuclear Medicine* ***49****(4):1052-1059*
85. *Gambhir S, Prashanth A, Pradhan P, et al. (2016) Impact of PET-CT in soft-tissue sarcomas. Journal of Nuclear Medicine* ***57***
86. *Lieberman PH, Brennan MF, Kimmel M, Erlandson RA, Garin-Chesa P and Flehinger BY (1989) Alveolar soft-part sarcoma. A clinico-pathologic study of half a century. Cancer* ***63****(1):1-13*
87. *Portera CA, Jr., Ho V, Patel SR, et al. (2001) Alveolar soft part sarcoma: clinical course and patterns of metastasis in 70 patients treated at a single institution. Cancer* ***91****(3):585-91*
88. *Espat NJ, Bilsky M, Lewis JJ, Leung D and Brennan MF (2002) Soft tissue sarcoma brain metastases. Prevalence in a cohort of 3829 patients. Cancer* ***94****(10):2706-11*
89. *Damery S, Biswas M, Billingham L, Barton P, Al-Janabi H and Grimer R (2014) Patient preferences for clinical follow-up after primary treatment for soft tissue sarcoma: a cross-sectional survey and discrete choice experiment. Eur J Surg Oncol* ***40****(12):1655-61*

Section 3. Special aspects. Comments

3.1. Non-malignant entities that require imaging for therapy control:

- Desmoid fibromatosis, lipoblastoma, inclusion body fibromatosis, calcifying aponeurotic fibroma, Gardner fibroma require imaging for the control of therapy (among other entities, such as superficial fibromatosis or tenosynovial giant cell tumor (TSGCT)).

Comments:

The reason for control imaging of non-malignant soft tissue cases mainly lies in the fact that these lesions may recur even years after therapy. Lipoblastoma may have a recurrence rate of 13-46% which is usually due to incomplete excision. The recurrence can occur as late as 6 years after primary resection. Inclusion body fibromatosis has recurrence rates between 61% and 75%. Local recurrence of calcifying aponeurotic fibroma may occur years after initial excision and is observed in as many as 50% of cases. Although Gardner fibroma is benign, subsequent development of desmoid fibromatosis is observed in approximately 20% of cases [1] . For the diffuse type of TSGCT, a multicentre- study reported a recurrence rate of 44% at a median follow-up period of 54 months (IQR 27–97) in surgically treated cases, with a recurrence-free survival of 55% at 5 and 40% at 10 years [2]. A lower recurrence rate has been described for localized TSGCT, e.g., 7% after a mean follow-up of 51.1 months in the foot and ankle region [3]. A study on TSGCT of the tendon sheath of the hand, including patients without and with satellite lesions or extension into the joints found a recurrence rate of 16% at a mean follow-up of 38.7 months [4] .

The treatment of plantar fibromatosis includes conservative approaches and/or surgery; post-operative recurrence rates after different surgical procedures vary; therefore future studies with standardized outcome measurements including imaging surveillance have been considered necessary [5] . The same is true for palmar fibromatosis (Dupuytren disease, DD), where recurrence rates are high [6], especially in cases with DD diathesis [7] .

3.2. Surveillance algorithms for non-malignant entities

3.2.1. Desmoid type fibromatosis

- A watchful waiting approach for asymptomatic patients is recommended.
- MRI is preferred. It should include T2w and contrast enhanced sequences.

- First re-evaluation should be done within 8–12 weeks, then every 3 months in the first year, then every 6 months up to the fifth year, and yearly thereafter.

Comments:
Desmoid fibromatosis is a locally aggressive, non-metastasizing neoplasm [1] with infiltrative growth [8] and propensity for local recurrence. A watchful waiting approach for asymptomatic patients is recommended because of recurrence after resection, possibility of morbidity with extensive surgery and observations of spontaneous regression [1]. With the development of new medications, systemic therapy is increasingly playing a central role in the treatment of desmoid fibromatosis [9].

Desmoid fibromatosis growth behavior significantly relates to T2 signal [10].· Higher T2 signal and contrast enhancement is associated with desmoid progression and rapid growth [11, 12] . Decreases in volume and T2 hyperintensity reflect the good response of desmoid fibromatosis [13].

3.2.2. Surveilllance in cancer predisposition syndromes

- Whole-body MR imaging and whole-body FDG PET/CT are useful in patients with **cancer predisposition syndromes**. Whole-body MR imaging is preferable since the patients are not exposed to ionizing radiation.

Comments:
Cancer predisposition syndrome patients such as those with Li Fraumeni syndrome need to surveillance throughout life [14-16] . Whole-body MR imaging has shown to have sufficient sensitivity without exposing the patients to ionizing radiation. The latter is very important since the screening mostly starts in pediatric/adolescent patients [15-17].

4.2. Role of interventional radiology

The role of interventional radiology is expanding in different scenarios:

- In the case of oligometastatic disease, patients should be considered for local therapies.
- In the case of local recurrences, patients should be considered for local therapies.

- Percutaneous cryoablation can be considered in cases of desmoid tumors and dermatofibrosarcoma protuberans.
- MR-guided high-intensity focused ultrasound can be considered for desmoid tumours.
- Interventional radiological procedures have a role in tumor control in a palliative setting.

Comments:

Although prospective randomized studies of sarcoma ablation are lacking due to the rarity and heterogeneity of the disease, the studies rereviewed suggest that percutaneous thermal ablation should be considered alongside surgery and radiation therapy in situations where local control or palliation of a sarcoma is desired [18-23].

Percutaneous image-guided treatments with ablation technologies (radiofrequency ablation, cryotherapy, microwaves ablation, high intensity focused ultrasound) provide high rate of durable local control for small-sized malignant deposit in many organs including lung, liver and bones[21]..When compared with surgery, image-guided treatments provide a lower-morbidity option with excellent tolerance and preservation of long-term function with low damage to healthy parenchyma around the metastasis[21].

Desmoid tumors are rare tumors considered of intermediate dignity because they lack metastatic potential; however, they remain very aggressive from the point of view of locoregional proliferation and recurrence after treatment. Several authors have reported the effectiveness and safety of cryoablation in the local treatment and for the analgesic management of desmoid tumors [20, 24]. MR-guided high intensity focused has emerged as a non-invasive alternative to conventional therapies, showing promising results and safety, with significantly and durably reduced viable tumour volume and pain in desmoid tumours[25].

For follow-up of advanced and refractory extra-abdominal desmoid fibromatosis after focal cryo-treatment, mRecist criteria should be followed on T1-FS subtraction images to detect focal nodular recurrences at the periphery of the ghost tumor and avoid hemorragic products pitfall after cryotherapy. Short term paradoxical growth may be observed secondary to edema and first year high slope volume decrease pattern post cryoterapy follow up[26]. Abscopal effect phenomenon of immune response of nontreated cryo- and radiotherapy areas may be seen[27, 28]. In case of systemic therapy, radiomics is the current criterion[29].

Dermatofibrosarcoma protuberans is also another rare cutaneous soft tissue sarcoma with a low risk of metastasis but a high risk of local recurrence despite wide local excision. Percutaneous cryoablation has been described in patients with local recurrences [30].

Future studies and clinical trials are necessary to refine the role of interventional radiology therapies into the multidisciplinary care of these patients.

1. *WHO Classification of Tumours tE, (2020), Volume 3; chapter 1,2 and 4; Available from: https://publications.iarc.fr/Book-And-Report-Series/Who-Classification-Of-Tumours/Soft-Tissue-And-Bone-Tumours-2020*
2. *Mastboom MJL, Palmerini E, Verspoor FGM, et al. (2019) Surgical outcomes of patients with diffuse-type tenosynovial giant-cell tumours: an international, retrospective, cohort study. Lancet Oncol **20**(6):877-886*
3. *Siegel M, Bode L, Sudkamp N, et al. (2021) Treatment, recurrence rates and follow-up of Tenosynovial Giant Cell Tumor (TGCT) of the foot and ankle-A systematic review and meta-analysis. PLoS One **16**(12):e0260795*
4. *Kolisetty PV, Ali SS, Ahmad I, Sudhy IK, Prakash O and Kishore YR (2024) Giant Cell Tumor of the Tendon Sheath of the Hand: Analysis of Factors Impacting Recurrence. Indian J Plast Surg **57**(2):123-128*
5. *Fuiano M, Mosca M, Caravelli S, et al. (2019) Current concepts about treatment options of plantar fibromatosis: A systematic review of the literature. Foot Ankle Surg **25**(5):559-564*
6. *Walker EA, Petscavage JM, Brian PL, Logie CI, Montini KM and Murphey MD (2012) Imaging features of superficial and deep fibromatoses in the adult population. Sarcoma **2012**:215810*
7. *Hindocha S, Stanley JK, Watson S and Bayat A (2006) Dupuytren's diathesis revisited: Evaluation of prognostic indicators for risk of disease recurrence. J Hand Surg Am **31**(10):1626-34*
8. *Milos RI, Moritz T, Bernathova M, et al. (2015) Superficial desmoid tumors: MRI and ultrasound imaging characteristics. Eur J Radiol **84**(11):2194-201*
9. *Alcindor T (2024) Building the Evidence for Systemic Treatment in Desmoid Fibromatosis: Is a New Metronomic Regimen Another Layer in the Foundation? JCO Glob Oncol **10**:e2300488*
10. *Gondim Teixeira PA, Biouichi H, Abou Arab W, et al. (2020) Evidence-based MR imaging follow-up strategy for desmoid-type fibromatosis. Eur Radiol **30**(2):895-902*
11. *Rosa F, Martinetti C, Piscopo F, et al. (2020) Multimodality imaging features of desmoid tumors: a head-to-toe spectrum. Insights Imaging **11**(1):103*
12. *Cassidy MR, Lefkowitz RA, Long N, et al. (2020) Association of MRI T2 Signal Intensity With Desmoid Tumor Progression During Active Observation: A Retrospective Cohort Study. Ann Surg **271**(4):748-755*
13. *Sheth PJ, Del Moral S, Wilky BA, et al. (2016) Desmoid fibromatosis: MRI features of response to systemic therapy. Skeletal Radiol **45**(10):1365-73*
14. *Kratz CP, Achatz MI, Brugieres L, et al. (2017) Cancer Screening Recommendations for Individuals with Li-Fraumeni Syndrome. Clin Cancer Res **23**(11):e38-e45*
15. *Greer MC (2018) Imaging of cancer predisposition syndromes. Pediatr Radiol **48**(9):1364-1375*
16. *Consul N, Amini B, Ibarra-Rovira JJ, et al. (2021) Li-Fraumeni Syndrome and Whole-Body MRI Screening: Screening Guidelines, Imaging Features, and Impact on Patient Management. AJR Am J Roentgenol **216**(1):252-263*
17. *Schafer JF, Granata C, von Kalle T, et al. (2020) Whole-body magnetic resonance imaging in pediatric oncology - recommendations by the Oncology Task Force of the ESPR. Pediatr Radiol **50**(8):1162-1174*
18. *Thompson SM, Schmitz JJ, Schmit GD, Callstrom MR and Kurup AN (2017) Image-Guided Thermal Ablative Therapies in the Treatment of Sarcoma. Curr Treat Options Oncol **18**(4):25*
19. *Bourcier K, Le Cesne A, Tselikas L, et al. (2019) Basic Knowledge in Soft Tissue Sarcoma. Cardiovasc Intervent Radiol **42**(9):1255-1261*
20. *Bouhamama A, Lame F, Mastier C, et al. (2020) Local Control and Analgesic Efficacy of Percutaneous Cryoablation for Desmoid Tumors. Cardiovasc Intervent Radiol **43**(1):110-119*

21. *Grilley-Olson JE, Webber NP, Demos DS, Christensen JD and Kirsch DG (2018) Multidisciplinary Management of Oligometastatic Soft Tissue Sarcoma. Am Soc Clin Oncol Educ Book **38**:939-948*
22. *Gutkin PM, von Eyben R, Chin A, et al. (2022) Local Control Outcomes Using Stereotactic Body Radiation Therapy or Surgical Resection for Metastatic Sarcoma. Int J Radiat Oncol Biol Phys **114**(4):771-779*
23. *Matsui JK, Jackson S, Fang J, et al. (2024) Effect of palliative radiation dose on symptom response in metastatic sarcomas. Clin Transl Radiat Oncol **48**:100803*
24. *Cazzato RL, Gantzer J, de Marini P, et al. (2022) Sporadic Desmoid Tumours: Systematic Review with Reflection on the Role of Cryoablation. Cardiovasc Intervent Radiol **45**(5):613-621*
25. *Ghanouni P, Dobrotwir A, Bazzocchi A, et al. (2017) Magnetic resonance-guided focused ultrasound treatment of extra-abdominal desmoid tumors: a retrospective multicenter study. Eur Radiol **27**(2):732-740*
26. *Auloge P, Garnon J, Robinson JM, et al. (2021) Percutaneous cryoablation for advanced and refractory extra-abdominal desmoid tumors. Int J Clin Oncol **26**(6):1147-1158*
27. *Aarts BM, Klompenhouwer EG, Rice SL, et al. (2019) Cryoablation and immunotherapy: an overview of evidence on its synergy. Insights Imaging **10**(1):53*
28. *Ko KWS, Chiang JB, Poon WL, Lai E and Garnon J (2021) Abscopal Effect After MRI-Guided Cryoablation of Multifocal Chest Wall Desmoid-Type Fibromatosis. Cardiovasc Intervent Radiol **44**(3):509-512*
29. *Crombe A, Kind M, Ray-Coquard I, et al. (2020) Progressive Desmoid Tumor: Radiomics Compared With Conventional Response Criteria for Predicting Progression During Systemic Therapy-A Multicenter Study by the French Sarcoma Group. AJR Am J Roentgenol **215**(6):1539-1548*
30. *Xu J, Li J, Zhou X, et al. (2014) Cryotherapy for local recurrent dermatofibrosarcoma protuberans: experience in 19 patients. Cryobiology **68**(1):134-8*

Additional information:

Comparison with guidelines of other societies/groups

Supp. Table S1. Recommendations by the ESSR in comparison with other societies/groups

ESSR 2024 (general strategy; for individualized strategies also see Table 2.2.2 and 2.4)

Year		1-3*	4-5	6- 10°
**High grade sarcoma**	Local imaging, chest imaging;	every 3-4 m	every 6 m	annually
**Low grade sarcoma***	Local imaging	every 4-6 m	annually	annually
				G1, R0 ev. patient initiated

*Baseline: not earlier than 3m. ° Continued after 10 years in WDLS (well-differentiated liposarcoma) and MLS (Myxoid Liposarcoma); for all, restarted after recurrence

ESSR 2015 [1, 2]

Year		1-3	4-5	6-
**High grade° sarcoma**	Local imaging, chest imaging;	every 3-4 m	every 6 m	annually
**Low grade sarcoma**	Local imaging[1]	every 4-6 m	every 6 m*	annually

ESMO-EURACAN-GENTURIS* Clinical Practice Guidelines (CPGs) [3]

Year		1-3	4-5	6-
**Intermediate-/high grade° sarcoma**	local MRI (extremities, superficial trunk)<br>Chest CT (lung metastases)	every 3-4 m	every 6 m	annually
**Low grade sarcoma**		every 6 m	every 6 m*	annually

** European Society for Medical Oncology; European Reference Network for Rare Adult Solid Cancers; European Reference Network for Genetic Tumour Risk Syndromes)*

NCCN [4]

Year		Up to 2-3	4-5	6-
**Stage IA/IB (low grade)**	PMH, physical examination	every 3-6 m	annually	annually
	Consider imaging (local[12] + chest[3])	every 6-12 months		
**Stage II/III (resectable)**	PMH, physical examination, chest imaging; local imaging [4]	every 3-6 m	every 6 m	annually
**Stage IV**	PMH, physical examination, chest CT, local imaging[5]	every 2-6 m	every 6 m	annually

[1]primary site based on locoregional recurrence with MR with or without contrast, or CT, or, for small or superficial lesions, US by experienced sonographer; [2]ev. not required in sites easily followed by physical examination; after 10y, individualized; [3]x-ray or CT. Special entities: similar to our ESSR recommendations. [4] Local imaging: based on estimated
risk of locoregional recurrence (In situations where the area is easily followed by physical examination, imaging may not be required. After 10 years, the likelihood of developing a recurrence is small and follow-up should be individualized).

[5]In situations where the area is easily followed by physical examination, imaging may not be required.

ACR (imaging in malignant or aggressive primary soft tissue tumors) [5]

**High grade sarcoma**	**LR**	MRI area of interest without and with contrast<br>FDG-PET/CT may be problem-solving in equivocal other study	every 3-6 m for 5y, then every 6-12m or if symptomatic
	**Lung metastases:**	Chest CT without contrast; FDG-PET/CT can be good problem-solving tool, seems to emerge as primary diagnostic tool in many MSK tumors	Not scientifically established. ESMO or NCCN
**Low grade sarcoma**	**LR**		
	**Lung metastases:**	Chest CT without contrast; FDG-PET/CT may be problem-solving in equivocal other study	every 3-6 m for 5y, then every 6-12m
**Sarcoma**	**Osseous metastases**	Imaging usually not appropriate in asymptomatic, usually appropriate in symptomatic patients<br>FDG-PET/CT whole body^<br>99mTc bone scan whole body°<br>Whole body MRI good alternative*	

Abbreviations: FDG-PET/CT 18F-Fluorodeoxyglucose Positron Emission Tomography/Computerised Tomography

^ can be a good problem-solving tool in individual cases

° Useful screening tool. In cases of abnormal spine uptake, SPECT/CT can be used to better distinguish metastases from degenerative changes

* if available (superior sensitivity and diagnostic accuracy compared to FDG-PET/CT cost effective, no ionizing radiation exposure)

ACR (imaging in malignant or aggressive primary soft tissue tumors) [6]

**LR**	MRI of the area of interest without and with iv contrast usually appropriate<br>MRI of the area of interest without iv contrast usually appropriate<br>FDG-PET/CT may be appropriate<br>FDG-PET/MRI may be appropriate<br>CT of the area of interest with IV contrast may be appropriate<br>US of the area of interest may be appropriate (no agreement)
**Lung metastases (patients without LR or known metastases):**	Chest CT without contrast usually appropriate<br>FDG-PET/CT may be appropriate

<u>British Sarcoma Group (BSG) Prognosis and follow up for primary disease [7]</u>

**Intermediate or high grade sarcoma**	**LR**	every 3-4m for 2-3y, then every 6m for years 3-5 and then annually years 5-10
	**Lung metastases:**	CXR
**Low grade sarcoma**	**LR**	every 4-6 m for 2-3y, then annually
	**Lung metastases:**	CXR
**Sarcoma**	**Osseous metastases**	Imaging usually not appropriate in asymptomatic,<br>usually appropriate in symptomatic patients<br>99mTc bone scan whole body°<br>Whole body MRI good alternative*

<u>ESSR 2024: Whole-body staging in sarcoma: tissue-specific additional modalities</u>

PET scan for osseous metastases	Alveolar soft part sarcoma, Angiosarcoma, Leiomyosarcoma, Undifferentiated pleomorphic sarcoma, Dedifferentiated liposarcoma
Regional MRI/CT for lymph nodes	High grade rhabdomyosarcoma, Clear cell sarcomas, Epithelioid sarcoma, Angiosarcoma, Synovial sarcoma
Abdominal CT	Epithelioid sarcoma, Angiosarcoma, Leiomyosarcoma, Hemangiopericytoma, PEComa
Brain imaging	Alveolar soft part sarcoma with lung metastases, Clear cell sarcoma, Angiosarcoma
Whole body MRI	Myxoid liposarcoma
Whole body PET/CT	Consider entity-depedent

1. *Noebauer-Huhmann IM, Weber MA, Lalam RK, et al. (2015) Soft Tissue Tumors in Adults: ESSR-Approved Guidelines for Diagnostic Imaging. Semin Musculoskelet Radiol* ***19****(5):475-82*

Eur Radiol (2024) Noebauer-Huhmann IM, Vilanova JC, Papakonstantinou O, et al.

2. AMSOS www.amsos.at, https://amsos.at/leitlinien-links/.
3. *Gronchi A, Miah AB, Dei Tos AP, et al. (2021) Soft tissue and visceral sarcomas: ESMO-EURACAN-GENTURIS Clinical Practice Guidelines for diagnosis, treatment and follow-up(☆). Ann Oncol* ***32****(11):1348-1365*
4. *von Mehren M, Kane JM, Agulnik M, et al. (2022) Soft Tissue Sarcoma, Version 2.2022, NCCN Clinical Practice Guidelines in Oncology. J Natl Compr Canc Netw* ***20****(7):815-833*
5. *Roberts CC, Kransdorf MJ, Beaman FD, et al. (2016) ACR Appropriateness Criteria Follow-Up of Malignant or Aggressive Musculoskeletal Tumors. J Am Coll Radiol* ***13****(4):389-400*
6. *Expert Panel on Musculoskeletal I, Stanborough R, Demertzis JL, et al. (2022) ACR Appropriateness Criteria(R) Malignant or Aggressive Primary Musculoskeletal Tumor-Staging and Surveillance: 2022 Update. J Am Coll Radiol* ***19****(11S):S374-S389*
7. *Dangoor A, Seddon B, Gerrand C, Grimer R, Whelan J and Judson I (2016) UK guidelines for the management of soft tissue sarcomas. Clin Sarcoma Res* ***6****:20*
